# Contemporary real-world treatment of cardiac transthyretin amyloidosis with tafamidis: a long-term multicentre study

**DOI:** 10.1093/eschf/xvag211

**Published:** 2026-07-30

**Authors:** Richard J Nies, Svenja Ney, Ingrid Kindermann, Yvonne Bewarder, Angela Zimmer, Fabian Knebel, Katrin Hahn, Sebastian Spethmann, Peter Luedike, Lars Michel, Tienush Rassaf, Maria Papathanasiou, Stefan Störk, Vladimir Cejka, Amin Polzin, Fabian Voß, Bernhard Unsöld, Christine Meindl, Michael Paulus, Ali Yilmaz, Bishwas Chamling, Caroline Morbach, Roman Pfister

**Affiliations:** Faculty of Medicine and University Hospital Cologne, Clinic III for Internal Medicine, University of Cologne, Kerpener Straße 62, Cologne 50937, Germany; Faculty of Medicine and University Hospital Cologne, Clinic III for Internal Medicine, University of Cologne, Kerpener Straße 62, Cologne 50937, Germany; Department of Internal Medicine III, University Hospital Saarland, Saarland University, Homburg 66421, Germany; Department of Internal Medicine III, University Hospital Saarland, Saarland University, Homburg 66421, Germany; Department of Internal Medicine III, University Hospital Saarland, Saarland University, Homburg 66421, Germany; Department of Internal Medicine II - Cardiology, Sana Klinikum Berlin-Lichtenberg, Berlin 10365, Germany; Deutsches Herzzentrum der Charité, Klinik für Kardiologie, Angiologie und Intensivmedizin, Charitéplatz 1, Berlin 10117, Germany; Klinik für Neurologie mit Experimenteller Neurologie, Charité Universitätsmedizin Berlin, Charitéplatz 1, Berlin 10117, Germany; Charité—Universitätsmedizin Berlin, corporate Member of Freie Universität Berlin and Humboldt-Universität zu Berlin, Charitéplatz 1, Berlin 10117, Germany; Amyloidosis Center Charité Berlin (ACCB), Charité—Universitätsmedizin Berlin, Charitéplatz 1, Berlin 10117, Germany; Deutsches Herzzentrum der Charité, Klinik für Kardiologie, Angiologie und Intensivmedizin, Charitéplatz 1, Berlin 10117, Germany; Charité—Universitätsmedizin Berlin, corporate Member of Freie Universität Berlin and Humboldt-Universität zu Berlin, Charitéplatz 1, Berlin 10117, Germany; Amyloidosis Center Charité Berlin (ACCB), Charité—Universitätsmedizin Berlin, Charitéplatz 1, Berlin 10117, Germany; Department of Internal Medicine/Cardiology and Intensive Care Medicine, Niels-Stensen Kliniken Marienhospital Osnabrück, Bischofsstraße 1, Osnabrück 49074, Germany; Department of Cardiology and Vascular Medicine, University Hospital Essen, University Duisburg-Essen, West German Heart and Vascular Center, Essen, Germany; Department of Cardiology and Vascular Medicine, University Hospital Essen, University Duisburg-Essen, West German Heart and Vascular Center, Essen, Germany; Department of Cardiology and Vascular Medicine, University Hospital Essen, University Duisburg-Essen, West German Heart and Vascular Center, Essen, Germany; Department of Cardiology and Vascular Medicine, University Hospital Essen, University Duisburg-Essen, West German Heart and Vascular Center, Essen, Germany; Department of Cardiology, University Hospital Frankfurt, Frankfurt 60590, Germany; Department Clinical Research and Epidemiology & Department Medicine I, University Hospital Würzburg, Comprehensive Heart Failure Center, Am Schwarzenberg 15, Würzburg 97078, Germany; Department Clinical Research and Epidemiology & Department Medicine I, University Hospital Würzburg, Comprehensive Heart Failure Center, Am Schwarzenberg 15, Würzburg 97078, Germany; Cardiovascular Research Institute Düsseldorf (CARID), Division of Cardiology, Pulmonology, and Vascular Medicine, Medical Faculty, University Düsseldorf, Düsseldorf, Germany; Cardiovascular Research Institute Düsseldorf (CARID), Division of Cardiology, Pulmonology, and Vascular Medicine, Medical Faculty, University Düsseldorf, Düsseldorf, Germany; Medical Clinic I, Cardiology and Angiology, Justus-Liebig-University Giessen, Klinikstrasse 33, Giessen 35392, Germany; Department of Internal Medicine II, University Hospital Regensburg, Franz-Josef-Strauss Allee 11, Regensburg 93042, Germany; Department of Internal Medicine II, University Hospital Regensburg, Franz-Josef-Strauss Allee 11, Regensburg 93042, Germany; Department of Internal Medicine II, University Hospital Regensburg, Franz-Josef-Strauss Allee 11, Regensburg 93042, Germany; Klinik für Kardiologie I, Sektion für Herzbildgebung, Universitätsklinikum Münster, Albert-Schweitzer-Campus 1, Münster 48149,Germany; Klinik für Kardiologie I, Sektion für Herzbildgebung, Universitätsklinikum Münster, Albert-Schweitzer-Campus 1, Münster 48149,Germany; Department of Internal Medicine B, University Medicine Greifswald and DZHK (German Centre for Cardiovascular Research), Partner Site Greifswald, Greifswald, Germany; Department Clinical Research and Epidemiology & Department Medicine I, University Hospital Würzburg, Comprehensive Heart Failure Center, Am Schwarzenberg 15, Würzburg 97078, Germany; Faculty of Medicine and University Hospital Cologne, Clinic III for Internal Medicine, University of Cologne, Kerpener Straße 62, Cologne 50937, Germany

**Keywords:** Cardiac amyloidosis, Cardiomyopathy, Heart failure, TTR, Transthyretin, Tafamidis

## Abstract

**Aims:**

Tafamidis was the first drug approved for transthyretin amyloid cardiomyopathy (ATTR-CM), but contemporary real-world evidence is lacking.

**Methods and results:**

This retrospective, multicentre study included ATTR-CM patients enrolled after commercial availability of tafamidis 61 mg in Germany (April 2020 to March 2021), with treatment initiation based on physician clinical judgement. Primary and secondary endpoints were all-cause mortality and the composite of all-cause mortality and/or heart failure (HF) hospitalization, respectively. The cohort comprised 329 patients (median age 80 [76–83] years, 85% male and 8% variant ATTR) and 80% received tafamidis. During a median follow-up of 39 [18–48] months, 21% of patients died and 42% reached the composite endpoint. Tafamidis-treated patients had a 3-year survival of 86% and an event-free survival of 63%. After propensity-score matching for age, New York Heart Association (NYHA) class, National Amyloidosis Centre (NAC) stage, and serum albumin, tafamidis was significantly associated with lower all-cause mortality (hazard ratio [HR] 0.28; 95%-confidence interval [CI] 0.13–0.61; *P* = .001) and fewer composite events (HR 0.56; 95%-CI 0.33–0.97; *P* = .039). In tafamidis-treated patients, primary and secondary endpoints were associated with advanced NAC stage (*P* < .031; *P* < .009), age ≥80 years (*P* = .022; *P* = .004), and serum albumin ≤42.5 g/L (*P* = .015; *P* < .001), whereas NYHA class ≥III predicted only the composite endpoint (*P* = .059; *P* < .001).

**Conclusions:**

This study provides long-term real-world outcome data for ATTR-CM patients treated with tafamidis based on physician clinical judgement. Adverse outcomes were strongly predicted by low serum albumin, advanced NAC stage, and older age, emphasizing the importance of early diagnosis and timely therapy of ATTR-CM.

## Graphical Abstract

**Graphical Abstract:**
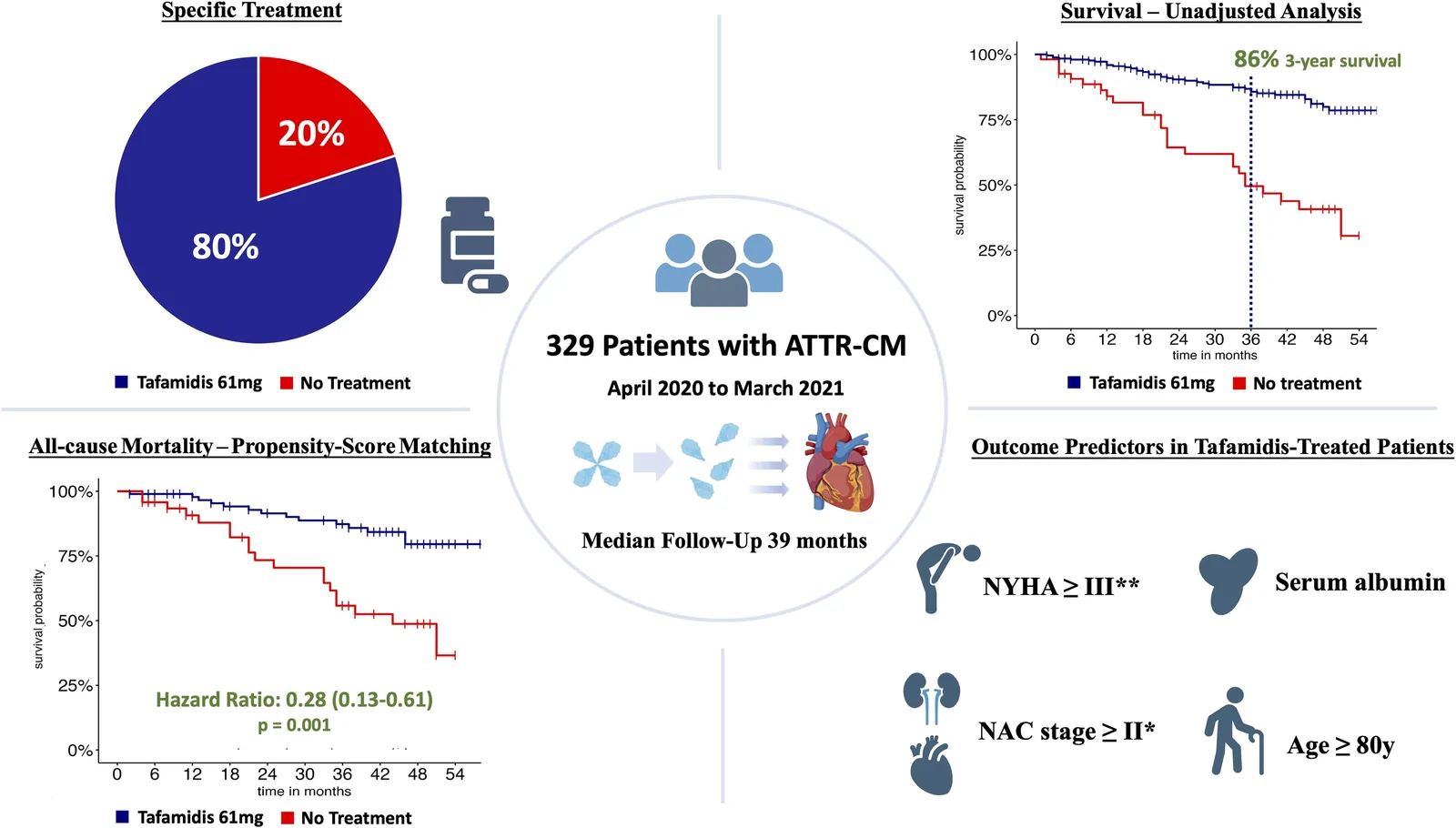
A total of 329 patients with transthyretin amyloid cardiomyopathy (ATTR-CM) from eight German tertiary centres, enrolled between April 2020 and March 2021 (covering the first year after approval of tafamidis 61 mg), were analysed for the primary endpoint of all-cause mortality. Treatment with tafamidis 61 mg was associated with a 3-year survival of 86%. After propensity-score matching (PSM) with untreated patients, tafamidis was associated with a 72% reduction in all-cause mortality. In tafamidis-treated patients, serum albumin ≤42.5 g/L, advanced National Amyloidosis Centre (NAC) stage, and age ≥80 years predicted both all-cause mortality (with regard to NAC risk stratification, only stage III was predictive of all-cause mortality [*]) and the composite endpoint, whereas higher New York Heart Association (NYHA) class was associated only with an increased risk of composite events [**].

## Introduction

Transthyretin amyloid cardiomyopathy (ATTR-CM) is a progressive disease caused by extracellular myocardial deposition of insoluble transthyretin (TTR) fibrils.^1^ Over the last decade, enhanced clinical awareness and advances in non-invasive diagnostics have led to an increase in the detection of ATTR wild-type (wt)-CM and diagnosis at earlier stages.^2–8^ Patient characteristics, disease course and outcome have undergone substantial changes and improvements over time.^9–12^

The pivotal ‘Transthyretin Amyloidosis Cardiomyopathy Clinical Trial’ (ATTR-ACT) provided evidence for the approval of tafamidis 61 mg as the first treatment of ATTR-CM independently of TTR genotype in Europe in 2020.^13^ However, patient enrolment in ATTR-ACT was more than 10 years ago which, together with patient selection bias inherent to randomized controlled trials (RCTs), limits the transferability of results to contemporary patients. This is of particular relevance for a potential comparison with drugs recently approved for the treatment of ATTR-CM based on pivotal trials enrolling patients from 2019 to 2021.^14,15^ Since head-to-head comparison trials between drugs are unlikely to be performed, indirect comparisons based on reasonably similar patient populations might provide helpful insight for physicians in their treatment decisions.

Although some real-world data exist for the use of tafamidis in ATTR-CM, these suffer from substantial limitations with respect to monocentric design, short follow-up, heterogeneous tafamidis dosing including doses not approved for ATTR-CM, a high proportion of patients with ATTR variant (v)-CM, and enrolment of non-contemporary or highly selected (compassionate use programmes or individual approval decisions by health insurance) patients.^16–21^ An additional major bias of several studies when assessing treatment effects of tafamidis was the imbalance in enrolment periods between treated and non-treated patients, which might result in overestimation of effect size.^17–19^

In this study we investigated contemporary real-world patients with ATTR-CM for whom the treatment decision was made after tafamidis approval and commercial availability, with the objectives of: (I) providing a benchmark for the long-term outcomes of contemporary ATTR-CM patients treated with tafamidis, (II) comparing long-term outcomes of patients treated with and without tafamidis, and (III) evaluating clinical risk markers in contemporary tafamidis-treated patients.

## Methods

### Study population

This retrospective, multicentre, observational registry study (ATTRreal-y1) included patients diagnosed with ATTR-CM who presented to participating tertiary care cardiology centres between 1 April 2020, and 31 March 2021. Accordingly, the enrolment period exclusively covered the first year following the approval and commercial availability of tafamidis 61 mg for ATTR-CM treatment in Germany. The participating centres comprised the University Hospitals in Düsseldorf, Berlin, Essen, Homburg, Cologne, Münster, Regensburg, and Würzburg. ATTR-CM diagnosis was confirmed locally at each site in accordance with the European Society of Cardiology’s cardiac amyloidosis working group criteria.^1^ Diagnostic methods included endomyocardial biopsy, extracardiac biopsy supported by characteristic cardiac imaging findings, or non-invasive diagnosis through positive technetium-99m hydroxymethylene diphosphonate (99mTc-HDP) bone scintigraphy in the absence of monoclonal gammopathy. Patients who had received an ATTR-CM diagnosis before 1 April 2020, were eligible for inclusion, but their first evaluation within the enrolment period was defined as baseline. Individuals already undergoing therapy with gene-silencers or tafamidis meglumine 20 mg for hereditary polyneuropathy (PNP) were excluded. In the absence of clearly defined recommendations for starting disease-modifying treatment, initiation of tafamidis 61 mg was determined by the treating physician’s clinical judgement, based on the published evidence available at that time and the patient’s overall clinical condition, with the intention of providing clinical benefit.^1^ Data used for analysis were anonymized and provided by each institution. The study adhered to the ethical principles of the Declaration of Helsinki and received approval from the ethics committees of the Universities of Cologne (22-1090 retro) and Würzburg (2021101101 and 2021101102).

### Data collection

Patient data regarding baseline characteristics and follow-up were extracted from standard clinical documentation at each participating centre. Data collected included demographics, comorbidities, laboratory biomarkers, concurrent medications, electrocardiograms, and imaging parameters. ATTR-CM stage was determined using the National Amyloidosis Centre (NAC) staging system, based on N-terminal pro-B-type natriuretic peptide (NT-proBNP) and glomerular filtration rate (GFR), stratifying patients into stages I-III.^11^ Echocardiographic examinations—including assessments of interventricular septum thickness at end-diastole (IVSd) and systolic function—were performed following current recommendations.^22^

### Study endpoints

The primary study endpoint was all-cause mortality. The secondary endpoint was a composite of all-cause mortality and/or hospitalization due to heart failure (HF). Follow-up for both endpoints was part of routine clinical care, and survival status was obtained from institutional records and telephone interviews. Patients were considered lost to follow-up if no information could be obtained after the baseline visit, either from the patient or their primary care physician.

### Data analysis

Categorical variables are presented as percentages, while continuous variables are summarized as means with standard deviations (SD) or medians with interquartile ranges. Patients were subgrouped by tafamidis 61 mg treatment status. Categorical variables were compared using the χ^2^-test or Fisher’s exact test. Continuous variables were analysed with an unpaired *t*-test or a Mann–Whitney *U* test, depending on data distribution. Data normality was evaluated with the Kolmogorov–Smirnov and Shapiro–Wilk tests.

Univariable Cox proportional hazards regression for the primary and secondary endpoints was applied to clinical variables that differed significantly between patients treated with and without tafamidis to assess their potential impact on outcomes. Variables significant in univariable analysis were included in multivariable Cox regression models. Hazard ratios (HRs) with 95%-confidence intervals (CI) were reported. To adjust for baseline imbalances of clinical characteristics between treatment groups, propensity-score matching (PSM) with a caliper width of 0.2 was performed.^23^ Matching variables were selected based on clinical relevance and multivariable Cox regression models, and missing data were handled using multiple imputation across 30 datasets before matching. Love plots of the pooled imputed datasets were used to assess covariate balance, with a standardized mean difference (SMD) below 0.1 considered to indicate excellent balance. Due to group size imbalance and improved covariate balance, a 2:1 nearest neighbour matching (two treated vs. one untreated) was applied. Survival was illustrated with Kaplan–Meier curves, and differences between groups were assessed using the Log-rank test. The most representative imputation was chosen by minimizing Euclidean distance to the SMDs. PSM using this imputation yielded a representative Kaplan–Meier curve and an overview of patient characteristics. HRs were pooled across all imputations using Rubin’s Rules and are reported on the Kaplan–Meier plot.

Regarding predictor analyses among tafamidis-treated patients, NAC stage, New York Heart Association (NYHA) class, and age were selected *a priori* based on their established prognostic significance in ATTR-CM. Serum albumin was included as a predictor based on our previous findings and its established role as a marker of frailty and nutritional status.^24,25^ An age threshold of 80 years was applied for comparability with recent studies.^20^ For serum albumin, an optimal cut-off in relation to the primary endpoint was determined using a receiver operating characteristic (ROC) curve analysis.

A two-sided *P*-value <.05 was considered statistically significant. All statistical analyses were conducted on an intention-to-treat basis using IBM SPSS Statistics for Macintosh (v29.0.0.0) and RStudio (v2025.05.1 + 513). Multiple imputation was conducted using the *mice* package, and PSM was performed with *MatchIt.* ROC curve analyses were performed in R using the *pROC* (v1.18.0) and *ROCR* (v1.0-11) packages. Kaplan–Meier survival curves were generated with the *survival* (v3.3-1) and *survminer* (v0.4.9) packages. Additional figures were created in Microsoft PowerPoint (v16.88.1) for Macintosh.

## Results

### Baseline characteristics

In total, we observed 329 patients with a median age of 80 [76–83] years; 85% (*n* = 280) were men, 8% (*n* = 26) had ATTRv-CM, and 80% (*n* = 262) received tafamidis (*Table 1*). Their median time since diagnosis of ATTR-CM was 3 [0–16] months. At baseline, 47% of patients reported dyspnoea classified as NYHA class ≥III, and 28% had a history of cardiac decompensation. According to the NAC staging system, 51% were in stage I, 33% in stage II, and 16% in stage III.^11^ The mean IVSd was 19 ± 4 mm, the median left ventricular ejection fraction (LVEF) was 54 [45–60]%, and the mean global longitudinal strain (GLS) was −10.6 ± 3.6%. Patients treated with tafamidis were younger, had lower NYHA classes and NAC stages, and higher serum albumin levels. The comparison of tafamidis-treated patients diagnosed within the past 12 months and those diagnosed ≥12 months earlier is shown in Supplementary Table S1. The median follow-up was 39 [18–48] months.

**Table 1:** Baseline characteristics of the ATTR-CM cohort, overall and stratified by treatment status

	ATTR-CM study cohort *n* = 329	Tafamidis 61 mg *n* = 262 (79.6%)	No specific therapy *n* = 67 (20.4%)	*P*
**Type of ATTR-CM**				
Wild-type; %	83.6	85.5	74.6	
Variant; %	7.9	6.9	11.9	.104
Not tested; %	8.5	7.6	13.4	
Time since initial diagnosis (months); Mdn [Q_1_;Q_3_]	3 [0; 16]	4 [0; 16]	2 [0; 14]	.162
≤12 months since initial diagnosis; %	71.4	71.0	73.1	.729
Follow-up time (months); Mdn [Q_1_;Q_3_]	39 [18; 48] (*n* = 313)	41 [21; 48] (*n* = 259)	22 [10; 43] (*n* = 54)	<.001
**Demographic data**				
Male patients; %	85.1	86.6	79.1	.122
Age (years); Mdn [Q_1_;Q_3_]	80 [76; 83]	79 [75; 82]	81 [76; 84]	.023
Age ≥80 years; %	50.2	46.6	64.2	.010
BMI (kg/m^2^); Mdn [Q_1_;Q_3_]	26.0 [24.1; 28.7] (*n* = 314)	26.0 [24.0; 28.7] (*n* = 253)	25.9 [24.3; 28.1] (*n* = 61)	.910
Modified BMI [(kg/m^2^)x(g/L)]; Mdn [Q_1_;Q_3_]	1125 [1030; 1250] (*n* = 203)	1155 [1031; 1259] (*n* = 156)	1086 [1016; 1234] (*n* = 47)	.206
Vital parameters				
SBP (mmHg); M ± SD	134 ± 19 (*n* = 282)	134 ± 19 (*n* = 229)	132 ± 21 (*n* = 53)	.533
DBP (mmHg); M ± SD	77 ± 12 (*n* = 282)	77 ± 11 (*n* = 229)	79 ± 14 (*n* = 53)	.662
HRa (1/min); M ± SD	73 ± 14 (*n* = 317)	72 ± 14 (*n* = 252)	72 ± 14 (*n* = 65)	.545
Symptoms				
NYHA class				
I; %	12.2 (*n* = 294)	11.1 (*n* = 235)	16.9 (*n* = 59)	
II; %	40.8 (*n* = 294)	43.8 (*n* = 235)	28.8 (*n* = 59)	
III; %	44.6 (*n* = 294)	44.3 (*n* = 235)	45.8 (*n* = 59)	.003
IV; %	2.4 (*n* = 294)	0.9 (*n* = 235)	8.5 (*n* = 59)	
History of cardiac decompensation; %	27.6 (*n* = 217)	29.2 (*n* = 168)	22.4 (*n* = 49)	.355
Comorbidities				
History of arterial hypertension; %	69.3 (*n* = 326)	68.0 (*n* = 259)	74.6	.291
Diabetes mellitus; %	20.0 (*n* = 325)	20.5 (*n* = 258)	17.9	.631
CAD; %	45.1 (*n* = 319)	45.8 (*n* = 253)	42.4 (*n* = 66)	.618
AF; %	61.5 (*n* = 317)	62.3 (*n* = 252)	58.5 (*n* = 65)	.570
History of CTS; %	38.4 (*n* = 318)	39.9 (*n* = 253)	32.3 (*n* = 65)	.260
Lumbar spinal stenosis; %	15.1 (*n* = 318)	17.0 (*n* = 253)	7.7 (*n* = 65)	.062
History of SAVR or TAVR; %	5.0 (*n* = 318)	4.4 (*n* = 252)	7.6 (*n* = 66)	.339
PNP; %	28.5 (*n* = 319)	31.2 (*n* = 253)	18.2 (*n* = 66)	.037
PM; %	16.5 (*n* = 315)	15.9 (*n* = 251)	18.8 (*n* = 64)	.588
Medication				
Diuretics; %	69.6 (*n* = 319)	70.4 (*n* = 253)	66.7 (*n* = 66)	.562
ACEi, ARB or ARNI; %	59.9 (*n* = 319)	60.5 (*n* = 253)	57.6 (*n* = 66)	.869
Beta-blocker; %	58.5 (*n* = 318)	57.5 (*n* = 252)	62.1 (*n* = 66)	.688
Oral anticoagulation; %	64.2 (*n* = 321)	63.5 (*n* = 255)	66.7 (*n* = 66)	.777
Biomarker				
Serum albumin (g/L); Mdn [Q_1_;Q_3_]	43 [41; 45] (*n* = 216)	43 [41; 45] (*n* = 164)	42 [40; 44] (*n* = 52)	.004
NT-proBNP (pg/mL); Mdn [Q_1_;Q_3_]	2633 [1336; 4446] (*n* = 308)	2590 [1369; 4313] (*n* = 252)	2836 [744; 6272] (*n* = 56)	.708
GFR (mL/min); Mdn [Q_1_;Q_3_]	57 [46; 75] (*n* = 312)	58 [49; 75] (*n* = 250)	54 [39; 74] (*n* = 62)	.088
HsTnT (ng/mL); Mdn [Q_1_;Q_3_]	0.044 [0.032; 0.070] (*n* = 202)	0.042 [0.032; 0.069] (*n* = 158)	0.047 [0.031; 0.071] (*n* = 44)	.637
Hb (g/dL); Mdn [Q_1_;Q_3_]	13.5 [12.6; 14.5] (*n* = 309)	13.4 [12.5; 14.5] (*n* = 248)	13.6 [12.6; 14.8] (*n* = 61)	.760
NAC stage				
I; %	50.8 (*n* = 303)	51.8 (*n* = 247)	46.4 (*n* = 56)	
II; %	33.3 (*n* = 303)	34.8 (*n* = 247)	26.8 (*n* = 56)	.042
III; %	15.8 (*n* = 303)	13.4 (*n* = 247)	26.8 (*n* = 56)	
Imaging				
LVEF (%); Mdn [Q_1_;Q_3_]	54 [45; 60] (*n* = 325)	54 [45; 60] (*n* = 260)	54 [43; 60] (*n* = 65)	.671
IVSd (mm); M ± SD	18.5 ± 3.6 (*n* = 325)	18.6 ± 3.4 (*n* = 260)	18.0 ± 4.3 (*n* = 65)	.208
E/e′; Mdn [Q_1_;Q_3_]	17 [12; 22] (*n* = 250)	16 [12; 22] (*n* = 213)	20 [13; 25] (*n* = 37)	.102
GLS (%); M ± SD	−10.6 ± 3.6 (*n* = 218)	−10.8 ± 3.3 (*n* = 163)	−10.2 ± 4.4 (*n* = 55)	.271
sPAP (mmHg); Mdn [Q_1_;Q_3_]	36 [30; 45] (*n* = 218)	36 [30; 45] (*n* = 188)	36 [30; 44] (*n* = 30)	.500
TAPSE (mm); Mdn [Q_1_;Q_3_]	17 [12; 21] (*n* = 314)	17 [13; 21] (*n* = 249)	16 [12; 22] (*n* = 65)	.940
Pericardial effusion; %	21.0 (*n* = 324)	20.8 (*n* = 259)	21.5 (*n* = 65)	.903

ACEi, angiotensin-converting enzyme inhibitor; AF, atrial fibrillation; ARB, angiotensin II receptor blocker; ARNI, angiotensin receptor neprilysin inhibitor; ATTR, transthyretin amyloidosis; ATTR-CM, transthyretin amyloid cardiomyopathy; BMI, body mass index; CAD, coronary artery disease; CTS, carpal tunnel syndrome; DBP, diastolic blood pressure; GFR, glomerular filtration rate; GLS, global longitudinal strain; Hb, haemoglobin; HRa, heart rate; hsTnT, high-sensitivity troponin T; IVSd, interventricular septum thickness at end-diastole; LVEF, left ventricular ejection fraction; M, mean; Mdn, median; NAC, National Amyloidosis Centre; NT-proBNP, N-terminal pro-B-type natriuretic peptide; NYHA, New York Heart Association; PM, pacemaker; PNP, polyneuropathy; Q, quartile; SAVR, surgical aortic valve replacement; SBP, systolic blood pressure; SD, standard deviation; sPAP, systolic pulmonary arterial pressure; TAPSE, tricuspid annular plane systolic excursion; TAVR, transcatheter aortic valve replacement; the ‘n’ within the columns indicates the number of patients for whom the respective variable was available.

### Long-term outcome by tafamidis treatment

Sixteen patients were lost to follow-up, including 13 without tafamidis treatment; thus, 313 patients were included in the endpoint analyses. During follow-up, 21% (*n* = 65) of the patients died and 42% (*n* = 130) experienced a composite endpoint (*Table 2*). Compared with patients without any events, those with all-cause mortality or a composite endpoint differed significantly in ATTR-CM subtype (wild-type vs. variant), age, NYHA class, use of diuretics and oral anticoagulants, serum albumin, NT-proBNP, GFR, high-sensitivity troponin T (hsTnT), NAC stage, LVEF, E/e′, GLS, tricuspid annular plane systolic excursion (TAPSE), and tafamidis treatment. Additionally, patients who died had significantly greater IVSd and more pacemakers. Those with the composite endpoint showed a lower modified body mass index, more prior decompensations, and higher rates of atrial fibrillation and coronary artery disease.

**Table 2:** Baseline characteristics of the ATTR-CM cohort with available follow-up, stratified by the primary endpoint of all-cause mortality and the secondary composite endpoint of all-cause mortality and/or hospitalization due to HF

	Primary endpoint			Secondary endpoint		
	No death *n* = 248 (79.2%)	All-cause mortality *n* = 65 (20.8%)	*P*	No events *n* = 183 (58.5%)	All-cause mortality and/or HF hospitalization *n* = 130 (41.5%)	*P*
Type of ATTR-CM						
Wild-type; %	84.3	80.0		80.9	86.9	
Variant; %	9.3	3.1	.010	11.5	3.1	.023
Not tested; %	6.5	16.9		7.7	10.0	
Time since initial diagnosis (months); Mdn [Q_1_;Q_3_]	3 [0; 14]	3 [0; 19]	.788	3 [0; 14]	4 [0; 17]	.691
≤12 months since initial diagnosis; %	71.8	67.7	.519	72.7	68.5	.418
Follow-up time (months); Mdn [Q_1_;Q_3_]	43 [22; 49]	21 [12; 35]	<.001	44 [22; 49]	33 [13; 44]	<.001
Demographic data						
Male patients; %	84.7	86.2	.767	84.7	85.4	.867
Age (years); Mdn [Q_1_;Q_3_]	79 [75; 82]	81 [78; 84]	.005	78 [72; 82]	80 [78; 84]	<.001
Age ≥80 years; %	44.8	61.5	.016	39.9	60.0	<.001
BMI (kg/m^2^); Mdn [Q_1_;Q_3_]	26.0 [24.1; 28.8] (*n* = 239)	26.4 [24.0; 28.7] (*n* = 60)	.740	26.1 [24.3; 29.0] (*n* = 175)	26.0 [23.9; 28.6] (*n* = 124)	.610
Modified BMI [(kg/m^2^) × (g/L)]; Mdn [Q_1_;Q_3_]	1154 [1051; 1278] (*n* = 143)	1105 [1011; 1192] (*n* = 51)	.057	1166 [1076; 1302] (*n* = 112)	1070 [988; 1194] (*n* = 82)	<.001
Vital parameters						
SBP (mmHg); M ± SD	134 ± 19 (*n* = 217)	133 ± 21 (*n* = 52)	.823	135 ± 19 (*n* = 156)	131 ± 20 (*n* = 113)	.121
DBP (mmHg); M ± SD	77 ± 12 (*n* = 217)	79 ± 13 (*n* = 52)	.133	77 ± 11 (*n* = 156)	77 ± 13 (*n* = 113)	.777
HRa (1/min); M ± SD	72 ± 13 (*n* = 240)	74 ± 15 (*n* = 62)	.374	72 ± 13 (*n* = 175)	74 ± 15 (*n* = 127)	.212
Symptoms						
NYHA class						
I; %	13.0 (*n* = 223)	10.5 (*n* = 57)		14.4 (*n* = 160)	10.0 (*n* = 120)	
II; %	44.4 (*n* = 223)	28.1 (*n* = 57)		49.4 (*n* = 160)	30.0 (*n* = 120)	
III; %	42.6 (*n* = 223)	49.1 (*n* = 57)	<.001	36.3 (*n* = 160)	54.2 (*n* = 120)	<.001
IV; %	0.0 (*n* = 223)	12.3 (*n* = 57)		0.0 (*n* = 160)	5.8 (*n* = 120)	
History of cardiac decompensation; %	26.1 (*n* = 161)	40.0 (*n* = 45)	.069	19.0 (*n* = 116)	42.2 (*n* = 90)	<.001
Comorbidities						
History of arterial hypertension; %	69.4 (*n* = 245)	69.2	.981	67.6 (*n* = 182)	71.9 (*n* = 128)	.420
Diabetes mellitus; %	19.7 (*n* = 244)	21.5	.738	19.2 (*n* = 182)	21.3 (*n* = 127)	.661
CAD; %	44.8 (*n* = 239)	46.9 (*n* = 64)	.764	40.3 (*n* = 176)	52.0 (*n* = 127)	.045
AF; %	60.3 (*n* = 237)	65.6 (*n* = 64)	.441	55.7 (*n* = 176)	69.6 (*n* = 125)	.014
History of CTS; %	38.1 (*n* = 239)	43.8 (*n* = 64)	.409	40.3 (*n* = 176)	37.8 (*n* = 127)	.654
Lumbar spinal stenosis; %	15.9 (*n* = 239)	14.1 (*n* = 64)	.718	15.3 (*n* = 176)	15.7 (*n* = 127)	.923
History of SAVR or TAVR; %	4.2 (*n* = 238)	7.8 (*n* = 64)	.326	5.1 (*n* = 176)	4.8 (*n* = 126)	.890
PNP; %	29.7 (*n* = 239)	28.1 (*n* = 64)	.805	29.0 (*n* = 176)	29.9 (*n* = 127)	.859
PM; %	14.0 (*n* = 236)	25.0 (*n* = 64)	.034	14.4 (*n* = 174)	19.0 (*n* = 126)	.279
Medication						
Diuretics; %	66.2 (*n* = 240)	87.3 (*n* = 63)	.001	60.6 (*n* = 175)	84.4 (*n* = 128)	<.001
ACEi, ARB or ARNI; %	57.1 (*n* = 240)	68.3 (*n* = 63)	.108	57.7 (*n* = 175)	61.7 (*n* = 128)	.483
Beta-blocker; %	57.3 (*n* = 239)	58.7 (*n* = 63)	.841	54.9 (*n* = 175)	61.4 (*n* = 127)	.255
Oral anticoagulation; %	61.0 (*n* = 241)	75.0 (*n* = 64)	.038	55.7 (*n* = 176)	75.2 (*n* = 129)	<.001
Biomarker						
Serum albumin (g/L); Mdn [Q_1_;Q_3_]	43 [42; 45] (*n* = 151)	42 [40; 44] (*n* = 56)	.001	44 [42; 45] (*n* = 119)	42 [40; 44] (*n* = 88)	<.001
NT-proBNP (pg/mL); Mdn [Q_1_;Q_3_]	2431 [1235; 4157] (*n* = 236)	3258 [1927; 6607] (*n* = 59)	.004	2074 [1005; 3681] (*n* = 178)	3698 [2026; 6263] (*n* = 117)	<.001
GFR (mL/min); Mdn [Q_1_;Q_3_]	59 [50; 75] (*n* = 235)	52 [38; 70] (*n* = 63)	<.001	60 [51; 77] (*n* = 176)	53 [41; 66] (*n* = 122)	<.001
HsTnT (ng/mL); Mdn [Q_1_;Q_3_]	0.041 [0.030; 0.063] (*n* = 147)	0.058 [0.038; 0.096] (*n* = 50)	<.001	0.040 [0.029; 0.059] (*n* = 118)	0.051 [0.037; 0.084] (*n* = 79)	<.001
Hb (g/dL); Mdn [Q_1_;Q_3_]	13.6 [12.6; 14.6] (*n* = 233)	13.2 [12.0; 14.4] (*n* = 63)	.155	13.6 [12.7; 14.6] (*n* = 174)	13.4 [12.2; 14.3] (*n* = 122)	.065
NAC stage						
I; %	53.7 (*n* = 231)	42.4 (*n* = 59)		60.3 (*n* = 174)	37.9 (*n* = 116)	
II; %	34.6 (*n* = 231)	28.8 (*n* = 59)	.005	28.2 (*n* = 174)	41.4 (*n* = 116)	<.001
III; %	11.7 (*n* = 231)	28.8 (*n* = 59)		11.5 (*n* = 174)	20.7 (*n* = 116)	
Imaging						
LVEF (%); Mdn [Q_1_;Q_3_]	54 [45; 60] (*n* = 244)	50 [40; 59]	.045	55 [50; 60] (*n* = 179)	49 [40; 58] (*n* = 130)	<.001
IVSd (mm); M ± SD	18.3 ± 3.6 (*n* = 244)	19.3 ± 3.3	.046	18.2 ± 3.7 (*n* = 179)	18.9 ± 3.3 (*n* = 130)	.083
E/e’; Mdn [Q_1_;Q_3_]	16 [12; 21] (*n* = 196)	21 [17; 26] (*n* = 44)	<.001	15 [12; 21] (*n* = 142)	19 [14; 24] (*n* = 98)	.003
GLS (%); M ± SD	−11.1 ± 3.5 (*n* = 150)	−9.4 ± 3.4 (*n* = 59)	.002	−11.4 ± 3.5 (*n* = 119)	−9.6 ± 3.3 (*n* = 90)	<.001
sPAP (mmHg); Mdn [Q_1_;Q_3_]	38 [30; 45] (*n* = 172)	35 [30; 45] (*n* = 38)	.945	35 [28; 44] (*n* = 119)	39 [30; 45] (*n* = 91)	.084
TAPSE (mm); Mdn [Q_1_;Q_3_]	17 [14; 21] (*n* = 235)	14 [11; 19] (*n* = 63)	.004	18 [14; 21] (*n* = 172)	15 [12; 19] (*n* = 126)	.001
Pericardial effusion; %	19.7 (*n* = 244)	28.1 (*n* = 64)	.142	19.0 (*n* = 179)	24.8 (*n* = 129)	.220

ACEi, angiotensin-converting enzyme inhibitor; AF, atrial fibrillation; ARB, angiotensin II receptor blocker; ARNI, angiotensin receptor neprilysin inhibitor; ATTR, transthyretin amyloidosis; ATTR-CM, transthyretin amyloid cardiomyopathy; BMI, body mass index; CAD, coronary artery disease; CTS, carpal tunnel syndrome; DBP, diastolic blood pressure; GFR, glomerular filtration rate; GLS, global longitudinal strain; Hb, haemoglobin; HF, heart failure; HRa, heart rate; hsTnT, high-sensitivity troponin T; IVSd, interventricular septum thickness at end-diastole; LVEF, left ventricular ejection fraction; M, mean; Mdn, median; NAC, National Amyloidosis Centre; NT-proBNP, N-terminal pro-B-type natriuretic peptide; NYHA, New York Heart Association; PM, pacemaker; PNP, polyneuropathy; Q, quartile; SAVR, surgical aortic valve replacement; SBP, systolic blood pressure; SD, standard deviation; sPAP, systolic pulmonary arterial pressure; TAPSE, tricuspid annular plane systolic excursion; TAVR, transcatheter aortic valve replacement; the ‘n’ within the columns indicates the number of patients for whom the respective variable was available.

**Figure 1:**
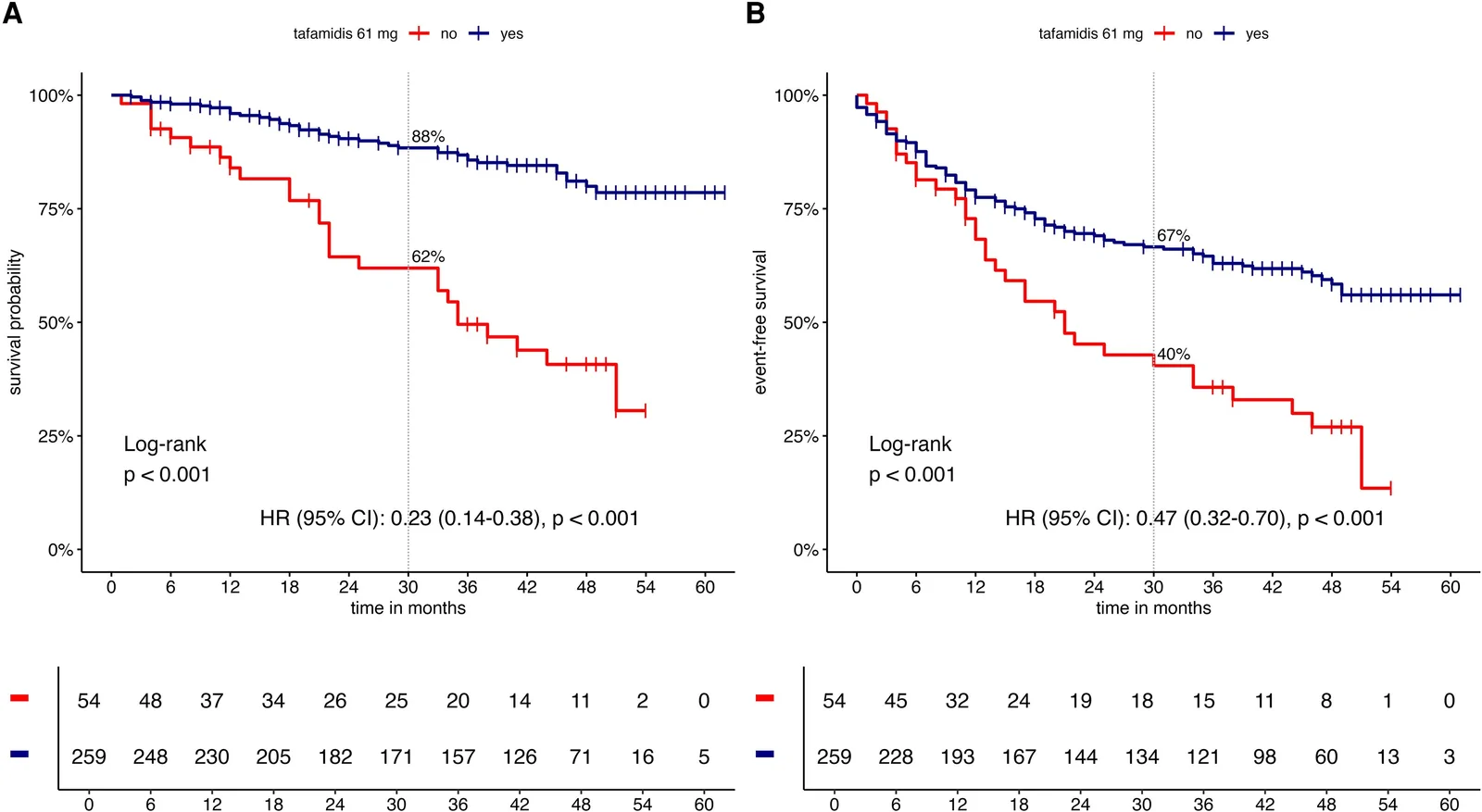
Kaplan–Meier curves for (*A*) the primary endpoint of all-cause mortality, and (*B*) the secondary composite endpoint of all-cause mortality and/or hospitalization due to HF, stratified by treatment status in the unmatched cohort

**Figure 2:**
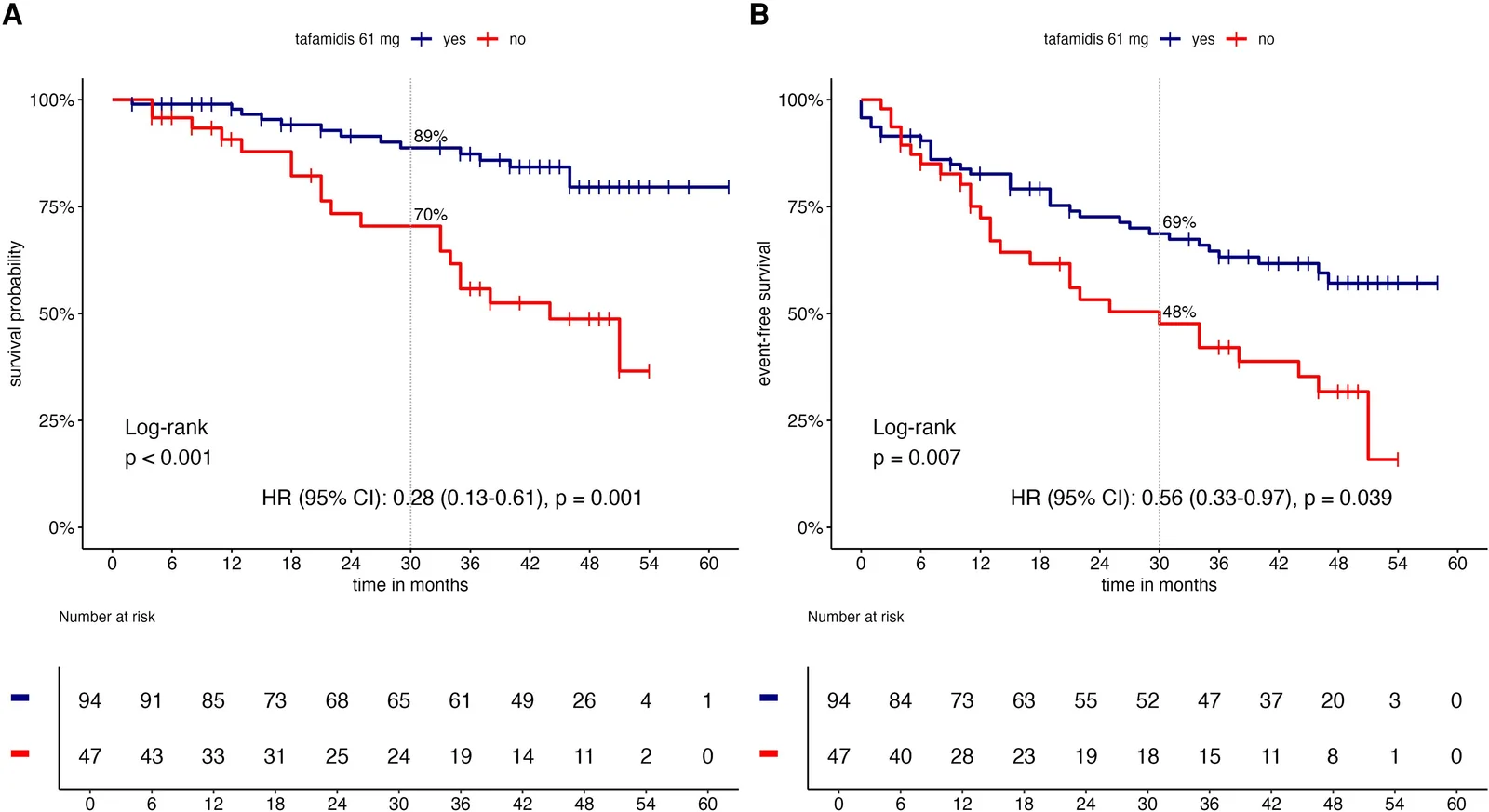
Representative Kaplan–Meier curves for (*A*) the primary endpoint of all-cause mortality, and (*B*) the secondary composite endpoint of all-cause mortality and/or hospitalization due to HF, stratified by treatment status in the propensity-score matched cohort (based on imputation 16)

At 1 and 3 years, the overall estimated survival of the total population was 94% and 80%, and the event-free survival was 76% and 59% (Supplementary Figures S1 and S2). Patients treated with tafamidis 61 mg had 1- and 3-year survival rates of 96% and 86% and event-free survival rates of 78% and 63%, respectively (*Figure 1*). Patients without tafamidis 61 mg treatment had survival rates of 84% and 50% at 1- and 3-year, and event-free survival rates of 68% and 36%. Tafamidis 61 mg was associated with lower all-cause mortality (HR 0.23; 95%-CI: 0.14–0.38; *P* < .001) and fewer composite events (HR 0.47; 95%-CI: 0.32–0.70; *P* < .001). No significant differences were observed between recently diagnosed tafamidis-treated patients and those diagnosed more than 12 months earlier (Supplementary Figure S3). Based on multivariable Cox regression analyses of variables differing between tafamidis-treated and untreated patients (Supplementary Tables S2 and S3), age, NYHA class, NAC stage, and serum albumin were used in the PSM which resulted in excellent covariate balance (Supplementary Figures S4–S6). In a representative imputed dataset, no significant differences in clinical characteristics between treatment groups were observed in the matched cohort (Supplementary Table S4). Cox regression in the PSM cohort showed that tafamidis treatment was associated with a 72% reduction in all-cause mortality (HR 0.28; 95%-CI: 0.13–0.61) and a 44% reduction in the composite endpoint (HR 0.56; 95%-CI: 0.33–0.97), compared with untreated patients (*Figure 2*). The survival benefit in tafamidis-treated patients persisted in both the ATTRwt-CM subgroup and in patients diagnosed within the past 12 months (Supplementary Figures S7–S10).

### Predictors of outcome in tafamidis-treated patients

**Figure 3:**
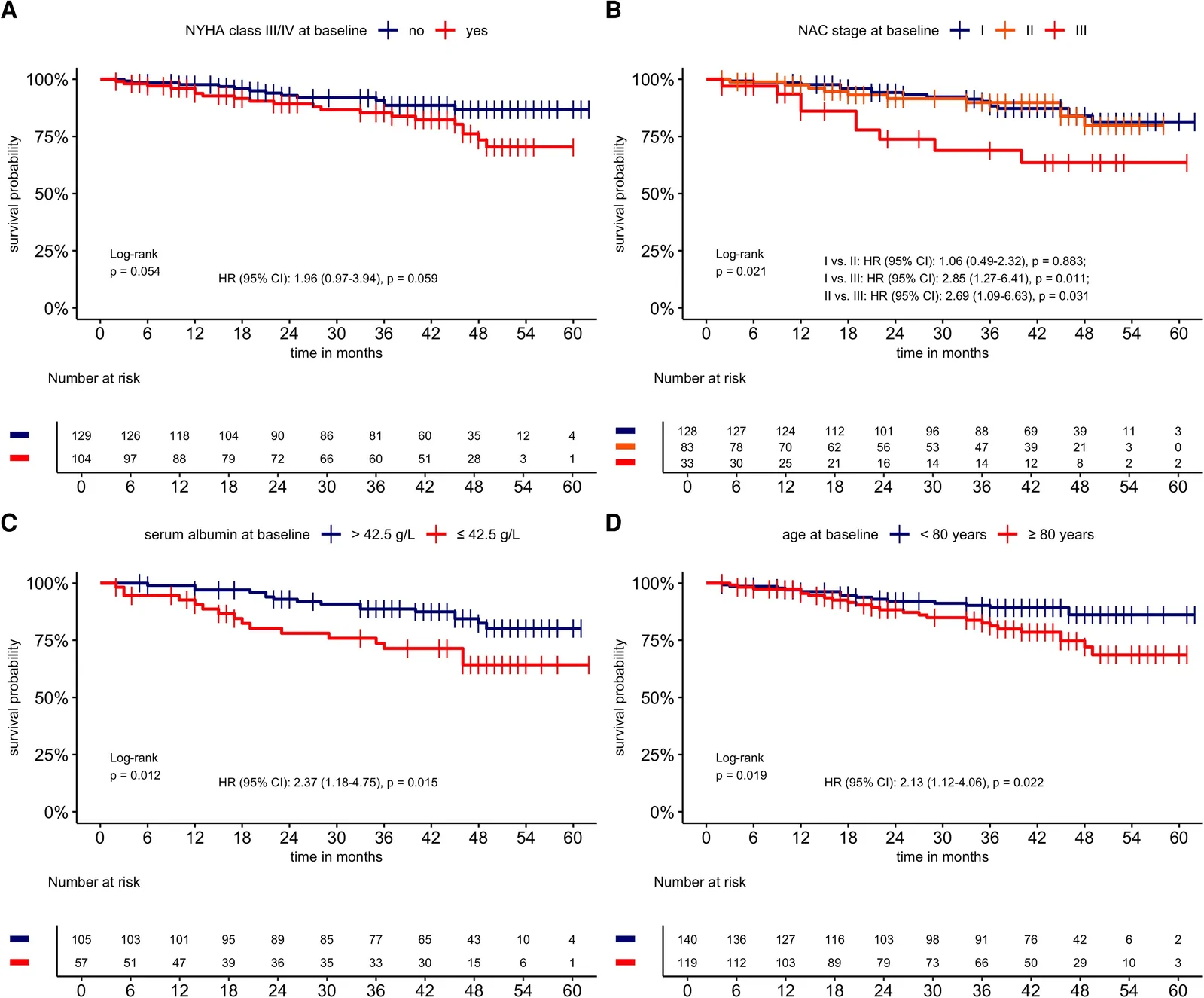
Kaplan–Meier curves for the primary endpoint of all-cause mortality among tafamidis-treated patients, stratified by (*A*) NYHA class, (*B*) NAC stage, (*C*) serum albumin, and (*D*) age

Among patients treated with tafamidis, higher NAC stage, lower serum albumin (≤42.5 g/L), and higher age (≥80 years) were associated with increased all-cause mortality, whereas the association of NYHA class ≥III was of borderline significance (*Figure 3*). Patients in NAC stage III had a significantly higher mortality risk than those in stages I and II, whereas stages I and II did not further differentiate risk.

**Figure 4:**
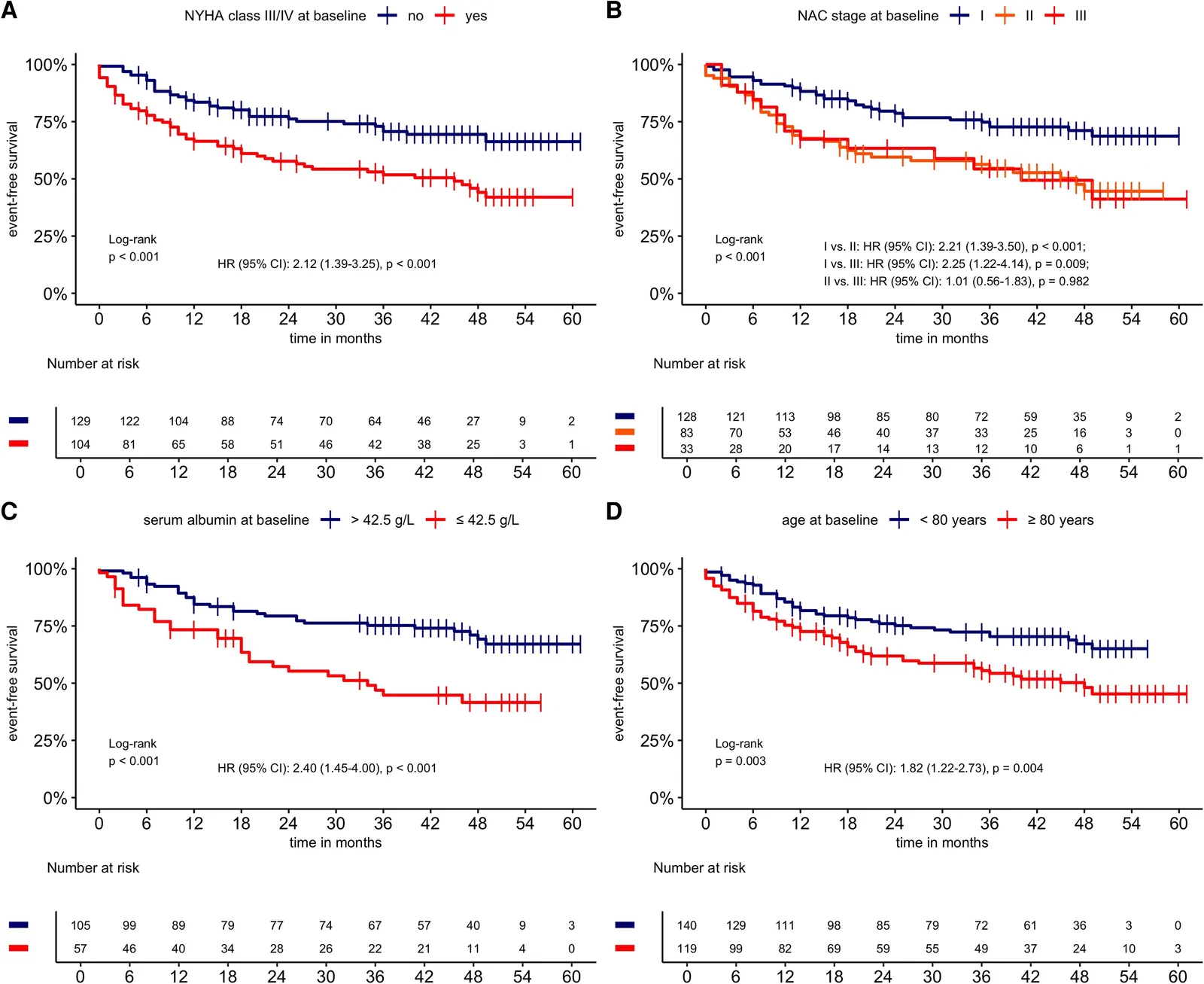
Kaplan–Meier curves for the secondary composite endpoint of all-cause mortality and/or hospitalization due to HF among tafamidis-treated patients, stratified by (*A*) NYHA class, (*B*) NAC stage, (*C*) serum albumin, and (*D*) age

NYHA class ≥III, higher NAC stage, serum albumin ≤42.5 g/L, and age ≥80 years significantly correlated with the composite endpoint (*Figure 4*). Patients in NAC stage I had a lower risk than those in stages ≥II, whereas stages II and III did not further discriminate risk.

## Discussion

The main findings from this analysis of a large contemporary real-world ATTR-CM cohort were:

1. Patients treated with tafamidis 61 mg exhibited a reasonably good outcome with survival and event-free survival of 96% and 78% at 1 year, and 86% and 63% at 3 years.
2. After adjusting for baseline imbalances, tafamidis treatment, based on physician clinical judgement, was associated with 72% and 44% reductions in all-cause mortality and the composite of all-cause mortality and/or HF hospitalization, with virtually identical results in ATTRwt patients and those diagnosed within the previous 12 months.
3. In tafamidis-treated patients, NAC stage, serum albumin, and age predicted both all-cause mortality and the composite endpoint, whereas NYHA class predicted only the composite endpoint.

### Real-world tafamidis evidence

Beyond smaller, mainly monocentric studies with inherent limitations of representativeness and statistical power, larger real-world reports have also shown major limitations in patient selection and follow-up time.^16,17,20,21,26^ In a recent analysis of the THAOS registry, more than 25% of tafamidis-treated and over 70% of untreated patients were enrolled between 2007 and 2018, before approval of tafamidis 61 mg, and the median follow-up of 2.2 years was relatively short.^17^ Furthermore, the participating THAOS centres were longstanding referral centres specialized in amyloidosis, and patients treated there may not be representative of broader amyloidosis care.^17^ Similarly, a recent report from five European heart centres included ATTRwt patients enrolled between 2009 and 2023 with a mean follow-up of 2.2 years.^20^ Most untreated patients were enrolled before 2018, while an unspecified proportion of treated patients were historical cases or received tafamidis via compassionate use or individual approval programmes, as commercial availability in these countries was only achieved at the end of 2021.^20^ In the large French ‘Healthcare European Amyloidosis Registry’, the tafamidis-treated group likewise comprised patients from early market access programmes between 2018 and 2021—prior to the official approval of tafamidis 61 mg—without reporting the exact share of these patients.^27^ This cohort was compared with historical untreated patients diagnosed before 2018.^27^ Finally, a study based on secondary data from electronic health records enrolled patients between 2019 and 2021 but reported follow-up of only 12 months.^16^ Overall, the existing literature on tafamidis use outside controlled trials is strongly biased by non-contemporary, highly selected cohorts and historical, imbalanced control groups, precluding valid estimation of outcomes and treatment effects of tafamidis.^16^ In contrast, our study provides a contemporary description of long-term real-world outcomes in patients with ATTR-CM enrolled after the approval and commercial availability of tafamidis 61 mg, with a median follow-up of more than 3 years.

### ATTR-CM outcome

In ATTR-ACT, 30-month survival was 71% with tafamidis treatment and 57% with placebo.^13^ In our real-world cohort, tafamidis-treated patients had a 30-month survival of 88%, whereas the untreated group reached 62%, both remarkably higher than in ATTR-ACT, suggesting an improvement in patient outcomes over time.^13^ Patients treated with and without tafamidis in ATTR-ACT had higher NT-proBNP levels than those in our cohort, suggesting more advanced disease at baseline.^13^ As declines in mortality among ATTR-CM patients correlate with the year of diagnosis independent of disease specific treatment, this substantial lead-time bias arising from earlier disease detection precludes the use of non-contemporary patient cohorts or the ATTR-ACT population as reference groups for comparative analyses.^13^

The ‘Acoramidis in Transthyretin Amyloid Cardiomyopathy’ (ATTribute-CM) study reported 30-month survival of 81% with the TTR stabilizer acoramidis, while the treatment arm of the ‘Vutrisiran in Patients with Transthyretin Amyloidosis with Cardiomyopathy’ (HELIOS-B) trial showed a slightly higher survival. Survival at 30 months in the corresponding placebo arms was 74% and slightly above 80%, respectively.^14,15^ Accordingly, treated patients in our cohort had higher survival than in RCTs, whereas the untreated patients experienced the lowest survival, probably reflecting selection bias related to decision-making for cost-intensive tafamidis treatment. Although both RCTs enrolled patients during a period similar to our real-world study, the interpretation and transferability of RCTs to real-world are limited by selective inclusion and exclusion criteria. RCTs usually exclude very advanced patients for reasons of futility while enriching patients at risk to achieve sufficient events and statistical power. For instance, HELIOS-B excluded ATTR-CM patients with NYHA class III and NAC stage III.^15^ This explains the low proportion of patients with NYHA class ≥III (<20%) compared with 45% in our unmatched tafamidis-treated real-world cohort, along with higher NT-proBNP levels and older age in our cohort. Despite this higher baseline risk, tafamidis-treated patients in our cohort exhibited superior survival compared with the RCT populations. However, direct comparisons should be made with caution, as our findings arise from an observational study with non-randomized treatment allocation. In clinical practice, treatment decisions rely on physician judgement based on a holistic clinical eyeballing in addition to NYHA class, biomarkers, and age. In conclusion, our findings underscore the importance of real-world data in complementing RCT evidence, demonstrating ongoing improvement in outcomes among ATTR-CM patients receiving diseasemodifying therapy, driven both by diagnosis at earlier disease stages and by physician-based patient selection.

### Outcome of tafamidis treatment

Even after PSM for key risk factors, tafamidis treatment remained associated with a markedly better outcome in our cohort, corresponding to a 72% reduction in all-cause mortality and a 44% reduction in the composite endpoint. In contrast, Maurer *et al*.^13^ reported a lower relative risk reduction in all-cause mortality of 30% in ATTR-ACT. The better outcome observed in the tafamidis group is likely overestimated, since residual confounding cannot be fully accounted for in a retrospective analysis. In particular, treatment decisions are currently based on few objective criteria and largely on overall clinical judgement. Consequently, patients with poor cardiac or extracardiac prognosis were most likely not treated with tafamidis. Consistently, our untreated group demonstrated worse absolute survival than placebo groups in contemporary RCTs, whereas our treated patients showed better absolute survival than treated patients in the respective trials.^14,15^ This supports the hypothesis of risk-adapted patient selection for tafamidis treatment.

### Predictors of adverse outcome in tafamidis-treated patients

Data on long-term outcomes and predictors of adverse outcome in real-world ATTR-CM patients treated with tafamidis 61 mg following its official approval remain limited, and risk scores such as the NAC staging system were developed in untreated populations.^9,11^ In our cohort, age, advanced NAC stage, and serum albumin were strong and independent predictors of all-cause mortality and the composite endpoint, whereas NYHA class III was not associated with all-cause mortality.

In historical cohorts before the advent of disease specific treatments, NYHA class was found to be an independent predictor of all-cause mortality.^12^ In contrast, more recent findings align with our results, indicating that among contemporary, specifically treated ATTR-CM patients, the prognostic value of NYHA class appears to be lower.^20^ Given that NYHA classification is inherently subjective and exhibits poor reproducibility—with interobserver agreement as low as 54% when differentiating class II from III—the usefulness of NYHA class for risk stratification may generally be overestimated.^28^ Furthermore, because a lower treatment benefit of tafamidis in NYHA class III patients was observed in ATTR-ACT, currently treated NYHA class III patients are likely positively selected—i.e. within the broad spectrum of NYHA III, only the healthier patients were selected for treatment—which may explain the limited prognostic stratification of NYHA classes.^13^

Biomarker-based risk stratification models are more objective than NYHA class and have shown prognostic value in treatment-naive patients.^9,11^ In our tafamidis-treated cohort, advanced NAC stage remained significantly associated with poorer outcome, but distinct NAC categories did not stratify risk levels as effectively as in historical cohorts.^11,19,20^ This may reflect that contemporary tafamidis-treated patients generally represent a lower-risk subgroup of all ATTR-CM patients, suggesting that NAC stage category cut-offs may require adaptation or further subcategorisation to improve stratification at lower risk. The benefits of such modifications have previously been reported in untreated patients with early-stage ATTR-CM.^29^

We also identified serum albumin as a predictor of adverse outcomes in patients treated with tafamidis 61 mg.^24^ Previous studies have linked lower serum albumin to frailty markers, including reduced handgrip strength, longer 5-meter walk time, and higher mortality risk, as well as to poorer outcomes in patients with general HF.^25,30^ Thus, serum albumin may serve as an easily assessable surrogate for frailty. Further studies might incorporate serum albumin into biomarker-based risk stratification models.

### Study limitations

As with any retrospective study, some data were incomplete. Serum albumin values were missing in approximately one-third of patients and were imputed, which could potentially attenuate the observed effect size due to regression to the mean; the true association of serum albumin with outcomes might be even stronger. There was an inherent selection bias in choosing patients for tafamidis treatment, likely favouring initiation in healthier individuals with fewer comorbidities and naturally better life expectancy. This was partially addressed using well-balanced PSM; however, adjustment for unmeasured prognostic confounders, such as additional comorbidities, frailty domains (e.g. poor mobility or weakness), and functional parameters including the six-minute walk test, was not possible. In the matched cohort, a significant difference in the presence of PNP persisted, but this did not affect survival outcomes, and PNP was more frequent in the tafamidis-treated subgroup. Regarding study endpoints, 16 patients were lost to follow-up, a small number relative to the total cohort. Since more than 80% of these patients were untreated, assuming high event rates in this subgroup would further support the observed treatment effect. Finally, hospitalizations due to HF may be underreported in routine clinical practice, which may have reduced the robustness of the corresponding analyses. Patient-reported outcome measures, including the Kansas City Cardiomyopathy Questionnaire, were not available.

### Conclusion

In this contemporary real-world cohort of ATTR-CM patients, those treated with tafamidis 61 mg showed favourable 3-year survival and event-free survival rates of 86% and 63%, respectively. These descriptive outcome data should be interpreted in light of the observational study design and non-randomized treatment allocation. After adjustment for measured baseline imbalances, tafamidis was associated with a 72% reduction in all-cause mortality and a 44% reduction in composite events. Established risk markers, such as NYHA class and NAC stage, might need adaptation of categories to the lower total risk in contemporary patients with disease specific treatment to allow reliable stratification. New, easily assessable biomarkers, like serum albumin, which reflect important clinical risk phenotypes such as frailty, may help improve risk classification in routine clinical practice.

## Supplementary Material

xvag211_Supplementary_Data

## Declarations

### Data Availability

No data were generated or analysed for this manuscript.

### Pre-registered Clinical Trial Number

None supplied.
