# Supplementary material for: Contemporary real-world treatment of cardiac transthyretin amyloidosis with tafamidis: a long-term multicentre study
Source: ESC Heart Fail. 2026 Jul 30;13(5):xvag211. doi: 10.1093/eschf/xvag211 (PMC13531469; doi:10.1093/eschf/xvag211)
Supplement: Supplementary file 1 [file xvag211_supplementary_data.pdf]

Contemporary Real-World Treatment of Cardiac Transthyretin Amyloidosis with Tafamidis: A Long-Term Multicentre Study

Richard J. Nies[1], Svenja Ney[1], Ingrid Kindermann[2], Yvonne Bewarder[2], Angela Zimmer[2], Fabian Knebel[3,4], Katrin Hahn[5,6,7], Sebastian Spethmann[4,6,7], Peter Luedike[8,9], Lars Michel[9], Tienush Rassaf[9], Maria Papathanasiou[9,10], Stefan Störk[11], Vladimir Cejka[11], Amin Polzin[12], Fabian Voß[12], Bernhard Unsöld[13,14], Christine Meindl[14], Michael Paulus[14], Ali Yilmaz[15], Bishwas Chamling[15,16], Caroline Morbach[11*], Roman Pfister[1*]

Short title: Long-Term Outcomes of Tafamidis-Treated ATTR-CM

Supplementary Material

[1] University of Cologne, Faculty of Medicine and University Hospital Cologne, Clinic III for Internal Medicine, Kerpener Straße 62, 50937 Cologne, Germany

[2] University Hospital Saarland, Department of Internal Medicine III, Saarland University, 66421 Homburg, Germany

[3] Department of Internal Medicine II - Cardiology, Sana Klinikum Berlin-Lichtenberg, 10365 Berlin, Germany

[4] Deutsches Herzzentrum der Charité, Klinik für Kardiologie, Angiologie und Intensivmedizin, Charitéplatz 1, 10117 Berlin, Germany

[5] Klinik für Neurologie mit Experimenteller Neurologie, Charité Universitätsmedizin Berlin, Charitéplatz 1, 10117 Berlin, Germany

[6] Charité – Universitätsmedizin Berlin, corporate member of Freie Universität Berlin and Humboldt-Universität zu Berlin, Charitéplatz 1, 10117 Berlin, Germany

[7] Amyloidosis Center Charité Berlin (ACCB), Charité - Universitätsmedizin Berlin, Charitéplatz 1, 10117 Berlin, Germany

[8] Niels-Stensen Kliniken Marienhospital Osnabrück, Department of Internal Medicine/Cardiology and Intensive Care Medicine, Bischofsstraße 1, 49074 Osnabrück, Germany

[9] University Hospital Essen, University Duisburg-Essen, West German Heart and Vascular Center, Department of Cardiology and Vascular Medicine, Essen, Germany

[10] University Hospital Frankfurt, Department of Cardiology, 60590 Frankfurt, Germany

[11] University Hospital Würzburg, Comprehensive Heart Failure Center, Department Clinical Research and Epidemiology & Department Medicine I, Am Schwarzenberg 15, 97078 Würzburg, Germany

[12] Cardiovascular Research Institute Düsseldorf (CARID), Division of Cardiology, Pulmonology, and Vascular Medicine, University Düsseldorf, Medical Faculty, Düsseldorf, Germany

[13] Medical Clinic I, Cardiology and Angiology, Justus-Liebig-University Giessen, Klinikstrasse 33, 35392 Giessen, Germany

[14] Department of Internal Medicine II, University Hospital Regensburg, Franz-Josef-Strauss Allee 11, 93042 Regensburg, Germany

[15] Klinik für Kardiologie I, Sektion für Herzbildgebung, Universitätsklinikum Münster, Albert-Schweitzer-Campus 1, Münster 48149, Germany

[16] Department of Internal Medicine B, University Medicine Greifswald and DZHK (German Centre for Cardiovascular Research), partner site Greifswald, Greifswald, Germany

* These authors contributed equally

Corresponding author:

Dr. med. Richard Nies

University of Cologne, Faculty of Medicine and University Hospital Cologne

Clinic III for Internal Medicine

Kerpener Straße 62

50937 Cologne, Germany

Phone: +49 221 478 76653

Fax: +49 221 478 32343

E-mail: richard.nies@uk-koeln.de

Figure S1: Overall all-cause mortality.

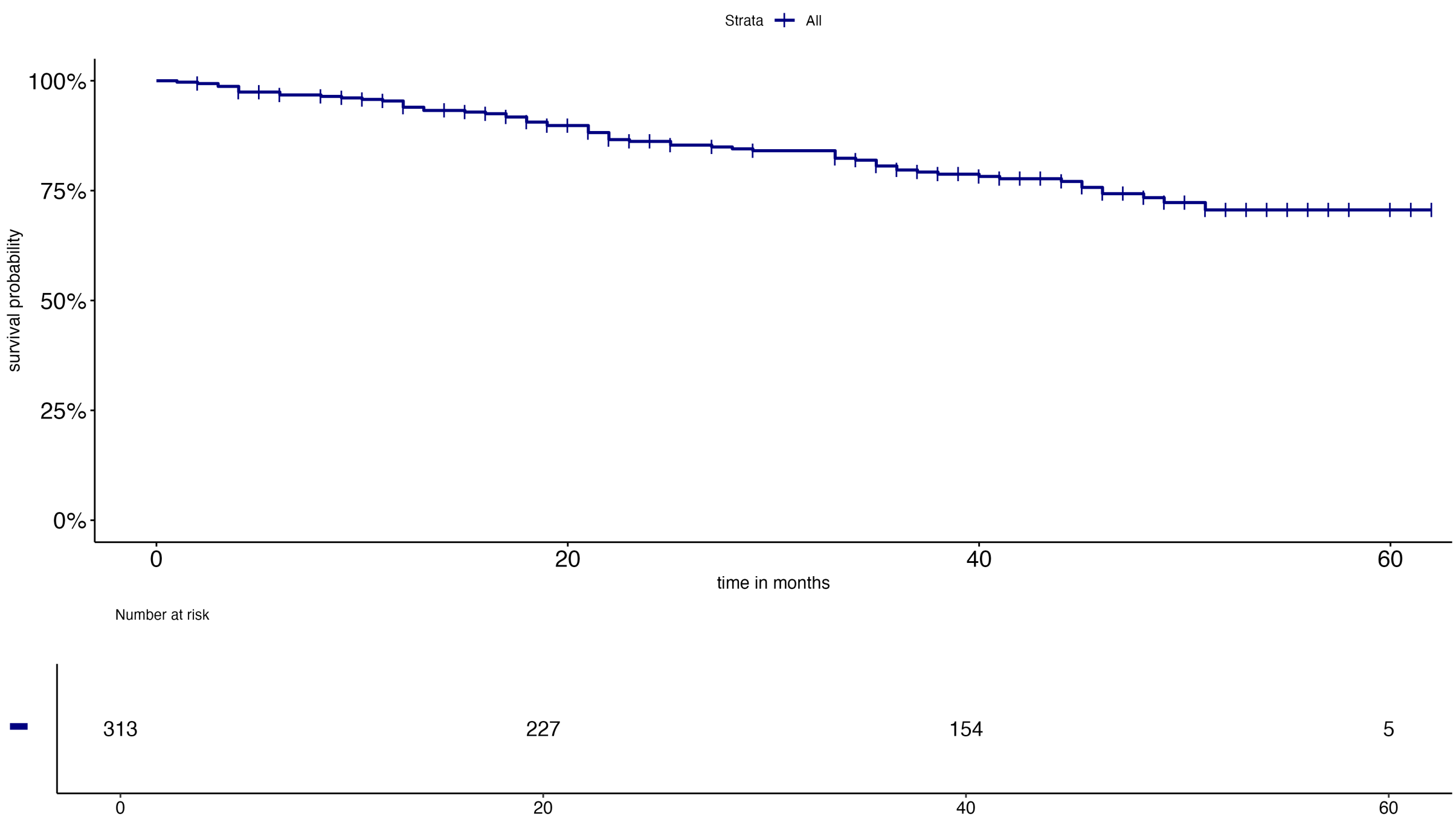

Figure S2: Overall composite of all-cause mortality and/or hospitalisation due to HF.

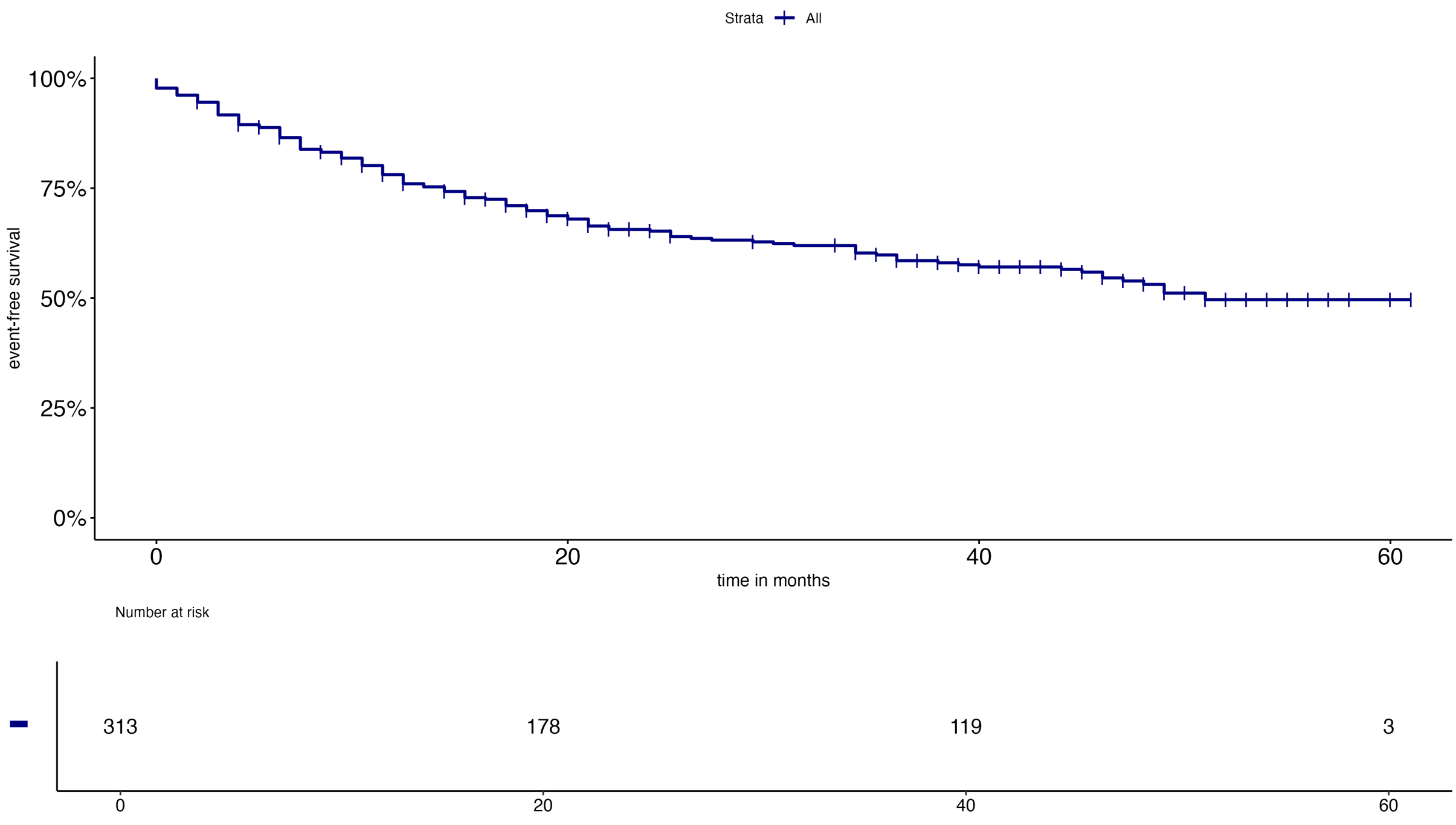

Figure S3: Kaplan-Meier curves for (**A**) the primary endpoint of all-cause mortality, and (**B**) the secondary composite endpoint of all-cause mortality and/or hospitalisation due to HF in tafamidis-treated patients stratified by time since ATTR-CM diagnosis.

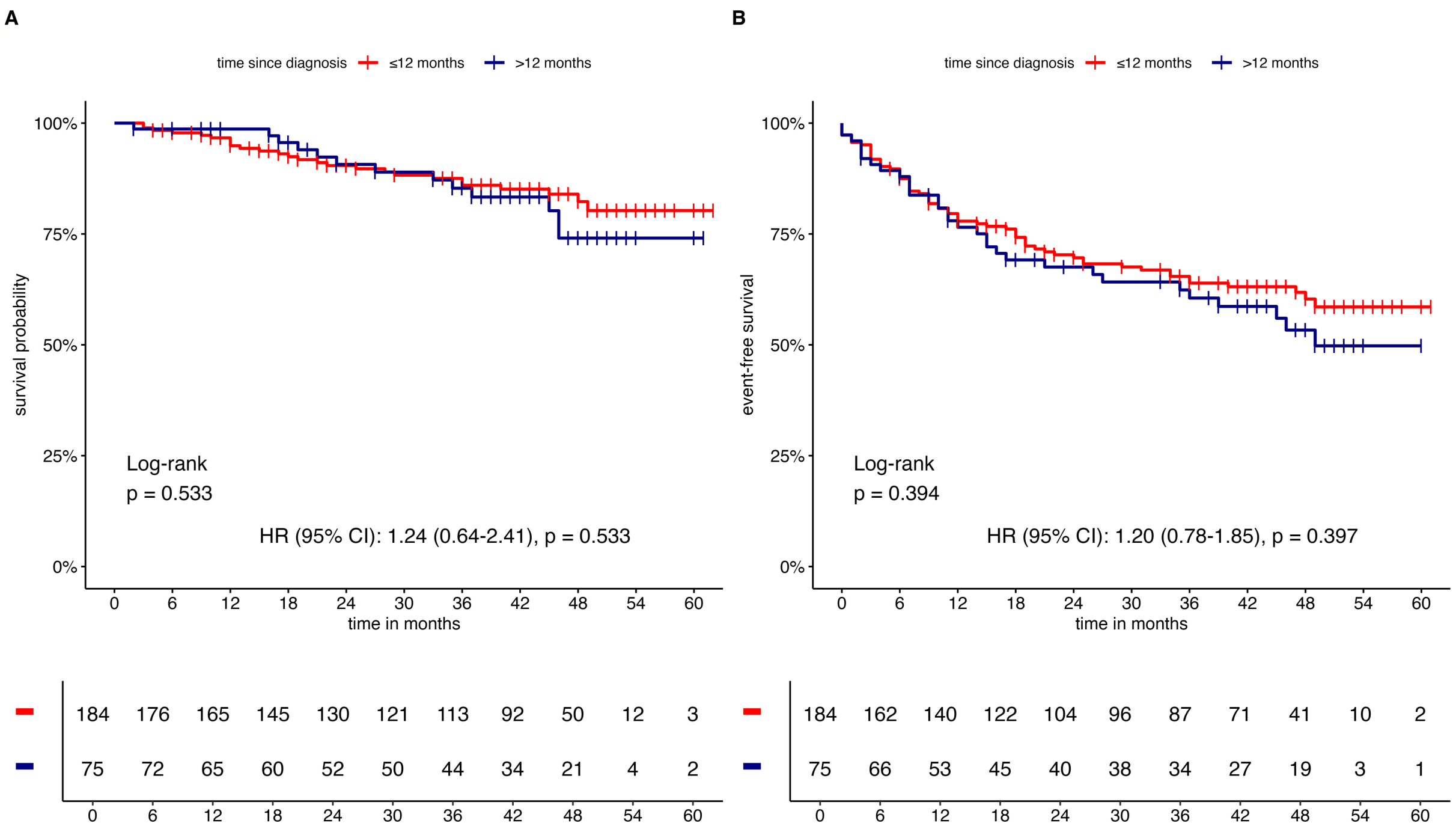

Figure S4: Love plot before propensity-score matching

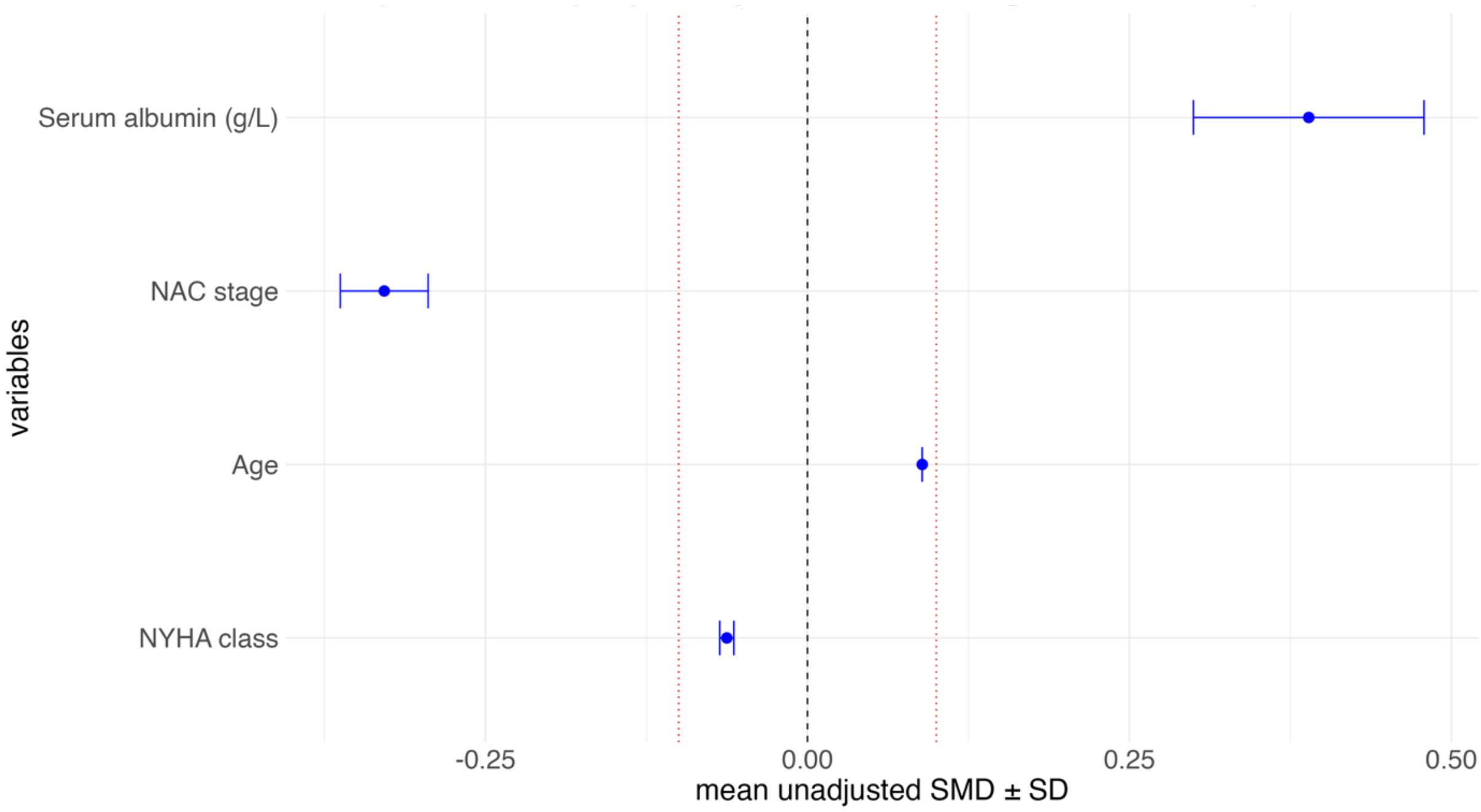

Figure S5: Love plot after propensity-score matching (1:1) across 30 imputations

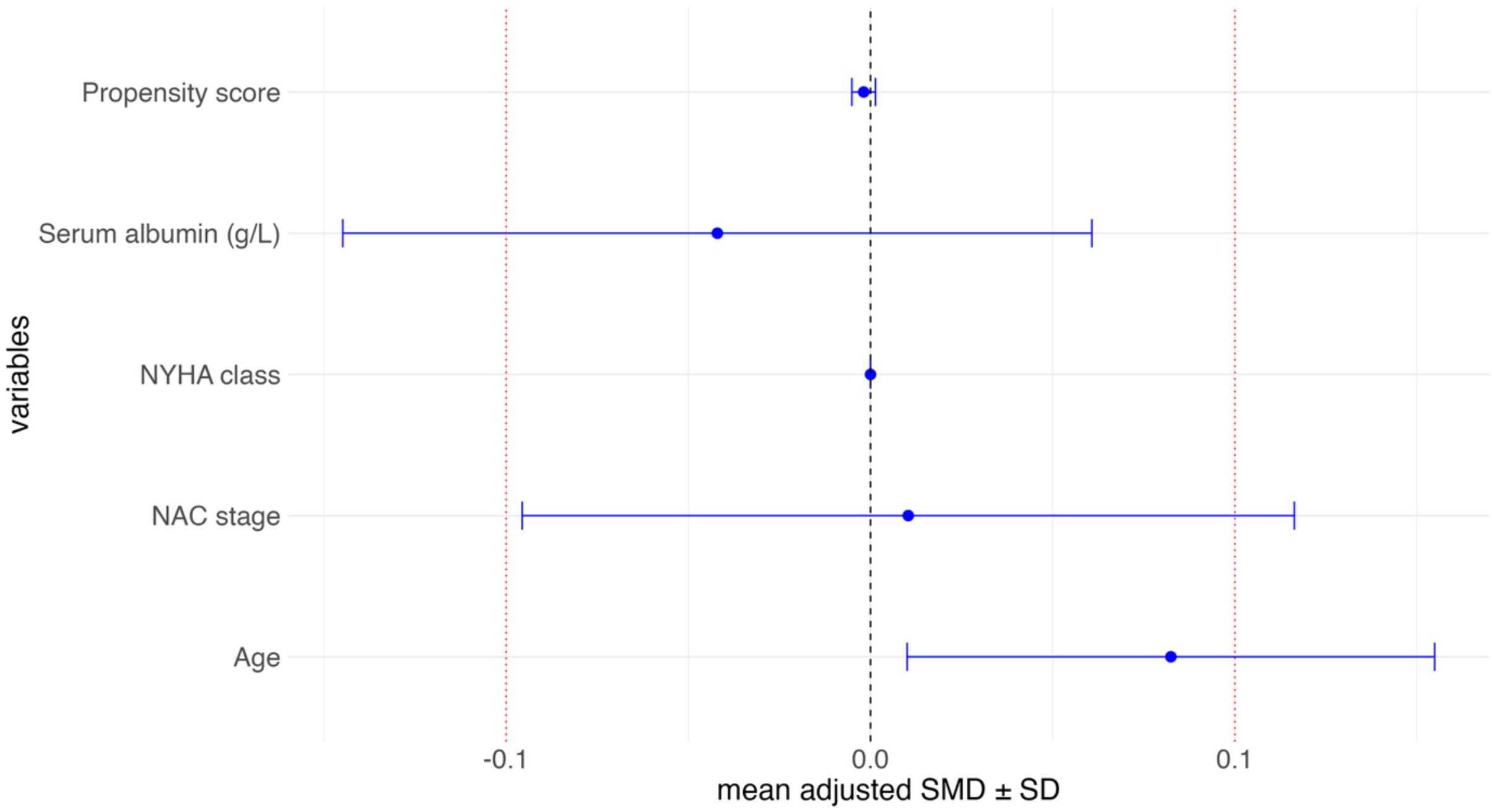

Figure S6: Love plot after propensity-score matching (2:1) across 30 imputations

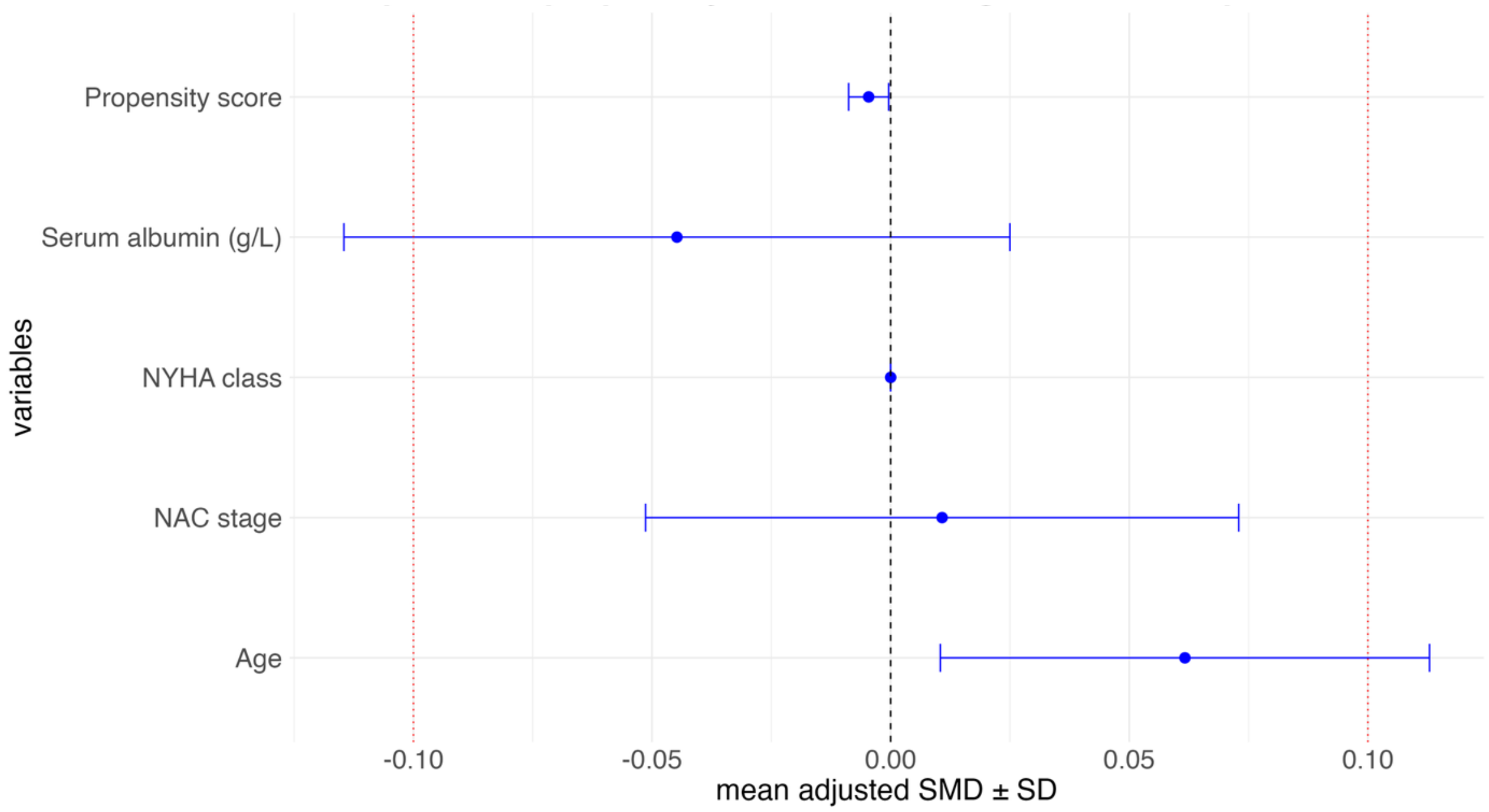

Figure S7: Love plot after propensity-score matching (2:1) across 30 imputations for patients with ATTRwt-CM.

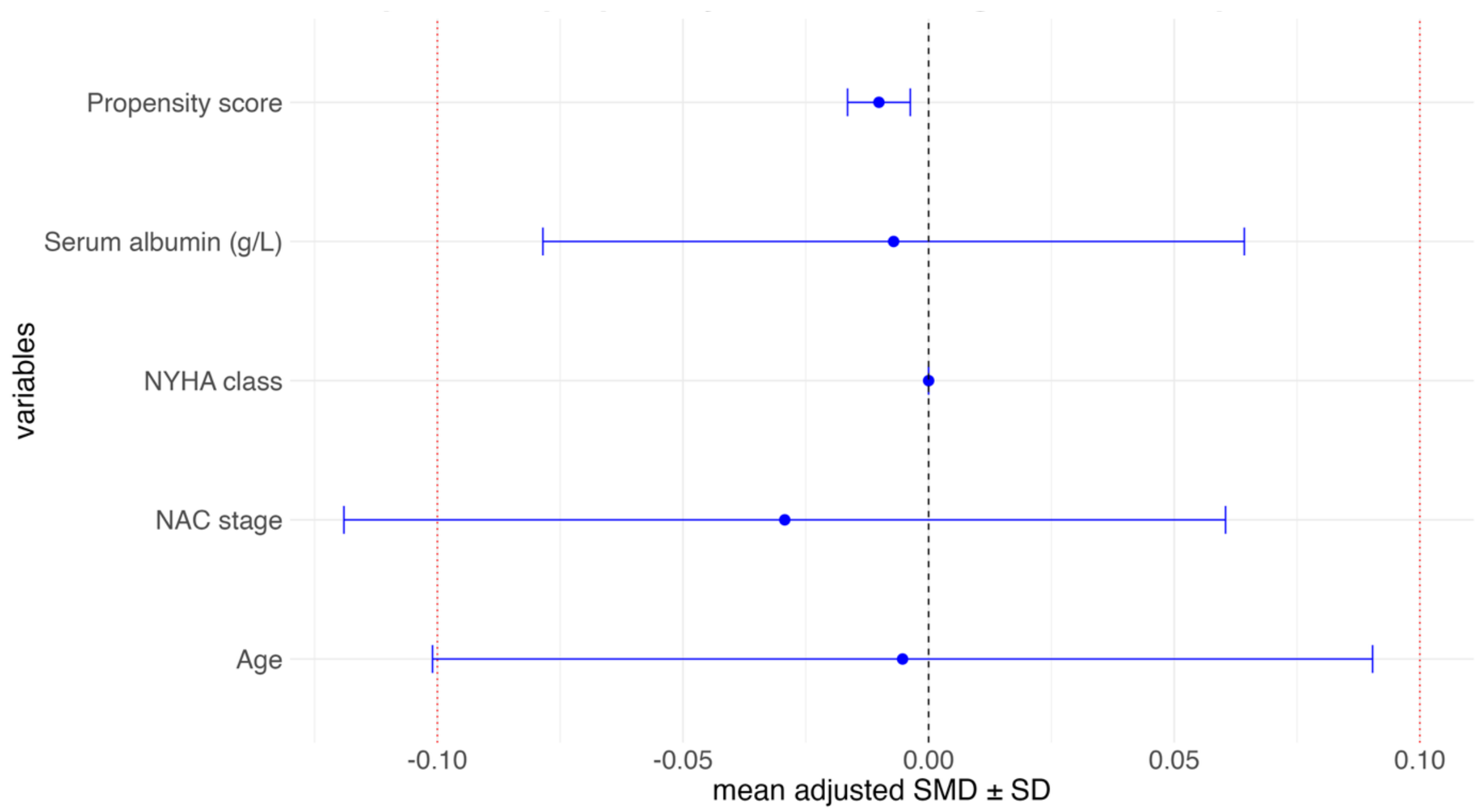

Figure S8: Representative Kaplan-Meier curves for (**A**) the primary endpoint of all-cause mortality, and (**B**) the secondary composite endpoint of all-cause mortality and/or hospitalisation due to HF, stratified by treatment status in the propensity-score matched cohort of patients with ATTRwt-CM.

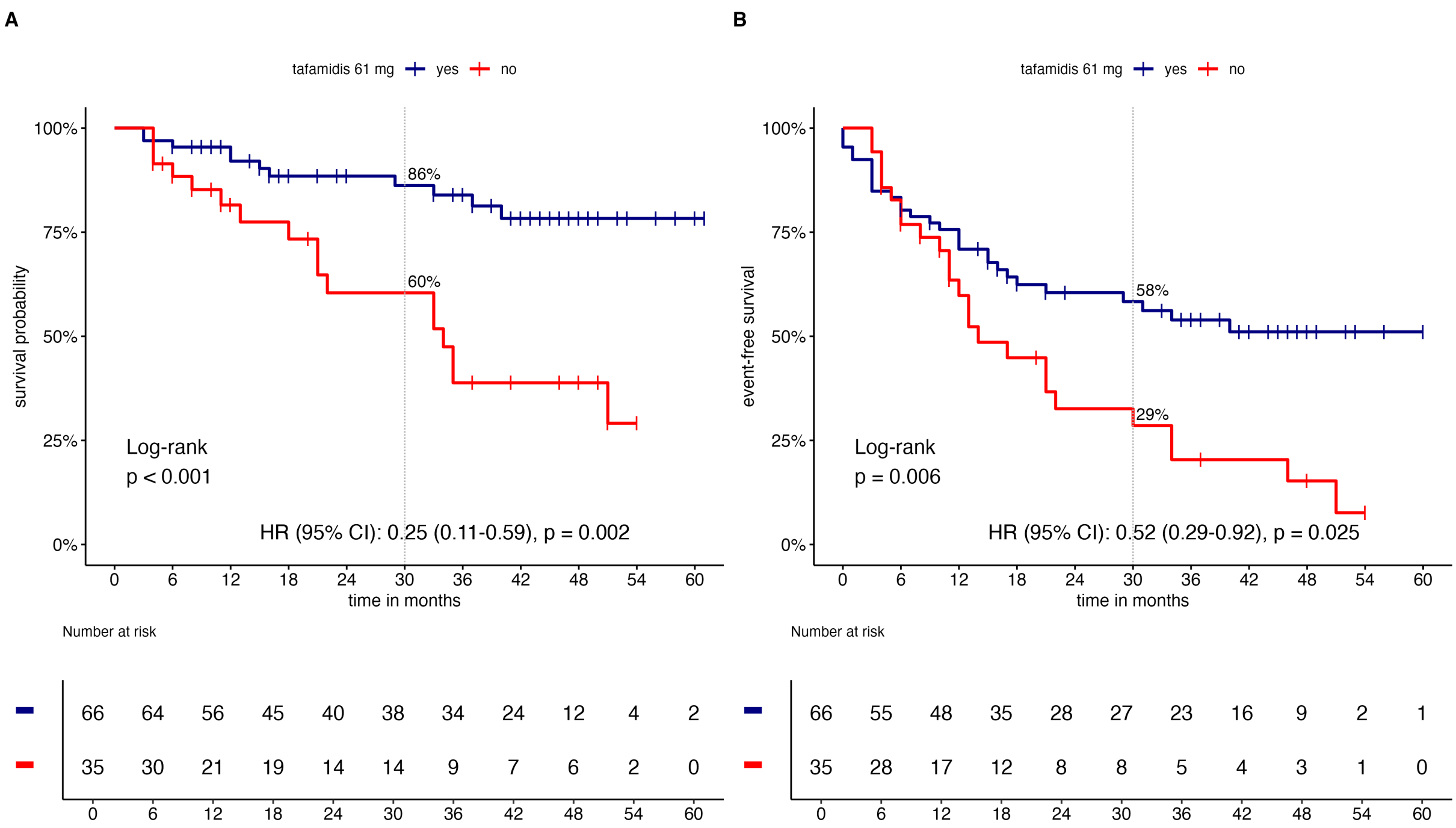

Figure S9: Love plot after propensity-score matching (2:1) across 30 imputations for patients with ATTR-CM and ≤ 12 months since initial diagnosis.

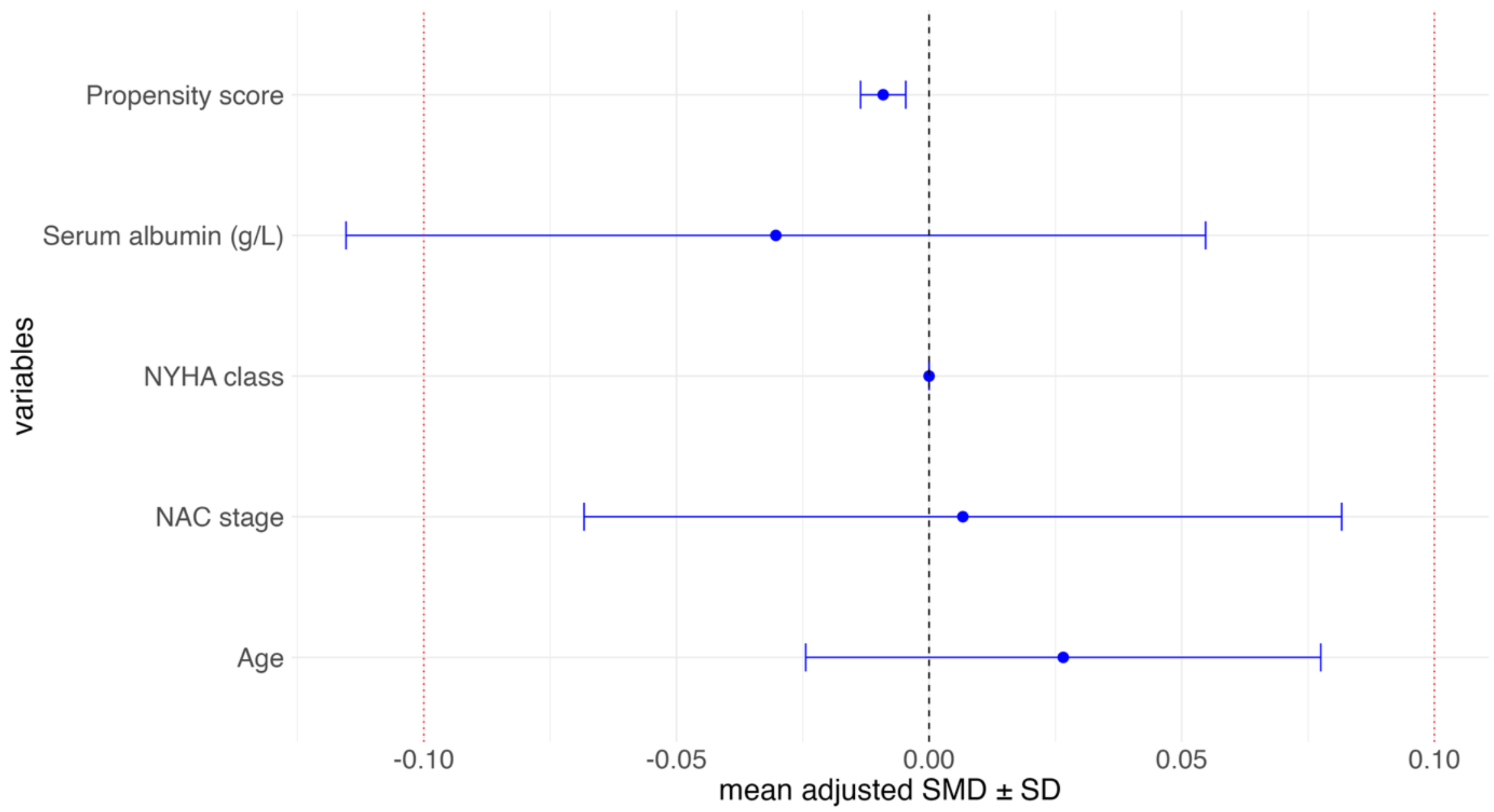

Figure S10: Representative Kaplan-Meier curves for (**A**) the primary endpoint of all-cause mortality, and (**B**) the secondary composite endpoint of all-cause mortality and/or hospitalisation due to HF, stratified by treatment status in the propensity-score matched cohort of patients with ATTR-CM and ≤ 12 months since initial diagnosis.

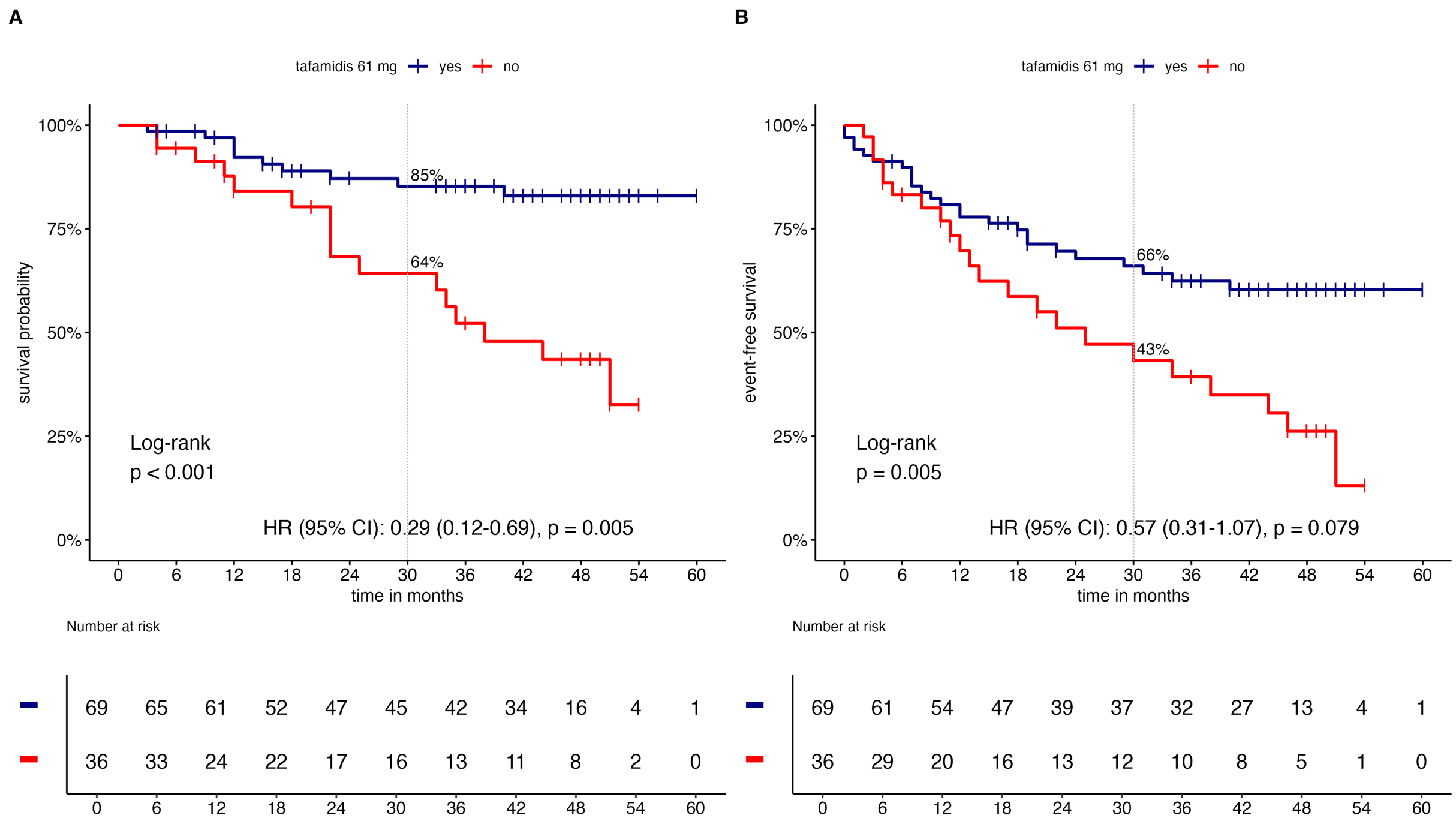

Table S1: Baseline characteristics of tafamidis-treated patients with ATTR-CM stratified by time since diagnosis.

	**Diagnosis ≤ 12 months** **n = 186 (71.0%)**	**Diagnosis > 12 months** **n = 76 (29.0%)**	**p**
Type of ATTR-CM			
wild-type; %	84.4	88.2	
variant; %	5.9	9.2	0.108
not tested; %	9.7	2.6	
Time since initial diagnosis (months); Mdn [Q_1;Q_3]	1 [0; 5]	23 [17; 34]	<0.001
Follow-up time (months); Mdn [Q_1;Q_3]	42 [19; 48] (n=184)	40 [22; 48] (n=75)	0.727
Demographic data			
Male patients; %	84.9	90.8	0.289
Age (years); Mdn [Q_1;Q_3]	79 [76; 82]	78 [72; 82]	0.302
Age ≥ 80 years; %	49.5	39.5	0.182
BMI (kg/m^2); Mdn [Q_1;Q_3]	25.9 [24.0; 28.7] (n=181)	26.4 [24.4; 28.7] (n=72)	0.605
Modified BMI [(kg/m^2)x(g/L)]; Mdn [Q_1;Q_3]	1,136 [1,031; 1,267] (n=113)	1,163 [1,042; 1,231] (n=43)	0.766
Vital parameters			
SBP (mmHg); M ± SD	134±18 (n=168)	133±21 (n=61)	0.757
DBP (mmHg); M ± SD	76±11 (n=168)	78±13 (n=61)	0.709
HRa (1/min); M ± SD	73±13 (n=182)	71±15 (n=70)	0.736
Symptoms			
NYHA class			
I; %	10.2 (n=172)	9.2 (n=63)	
II; %	42.5 (n=172)	31.6 (n=63)	
III; %	38.7 (n=172)	42.1 (n=63)	0.126
IV; %	1.1 (n=172)	0.0 (n=63)	
History of cardiac decompensation; %	26.0 (n=131)	40.5 (n=37)	0.123
Comorbidities			
History of arterial hypertension; %	66.3 (n=184)	72.0 (n=75)	0.457
Diabetes mellitus; %	20.2 (n=183)	21.3 (n=75)	0.975
CAD; %	47.3 (n=182)	42.3 (n=71)	0.564
AF; %	60.6 (n=180)	66.7 (n=72)	0.447
History of CTS; %	40.7 (n=182)	38.0 (n=71)	0.810
Lumbar spinal stenosis; %	17.6 (n=182)	15.5 (n=71)	0.833
History of SAVR or TAVR; %	4.4 (n=182)	4.3 (n=70)	1.000
PNP; %	28.0 (n=182)	39.4 (n=71)	0.108
PM; %	13.3 (n=180)	22.5 (n=71)	0.109
Medication			
Diuretics; %	66.3 (n=181)	80.6 (n=72)	0.037
ACEi, ARB or ARNI; %	61.0 (n=182)	59.2 (n=71)	0.901
Beta-blocker; %	55.2 (n=181)	63.4 (n=71)	0.302
Oral anticoagulation; %	63.7 (n=182)	63.0 (n=73)	1.000
Biomarker			
Serum albumin (g/L); Mdn [Q_1;Q_3]	44 [41; 45] (n=117)	43 [42; 45] (n=47)	0.739
NT-proBNP (pg/mL); Mdn [Q_1;Q_3]	2,309 [1,348; 3,924] (n=181)	3,521 [1,655; 5,954] (n=71)	0.009
GFR (mL/min); Mdn [Q_1;Q_3]	60 [51; 75] (n=179)	53 [44; 62] (n=71)	0.002
HsTnT (ng/mL); Mdn [Q_1;Q_3]	0.042 [0.032; 0.071] (n=112)	0.043 [0.035; 0.064] (n=46)	0.760
Hb (g/dL); Mdn [Q_1;Q_3]	13.2 [12.4; 14.3] (n=178)	13.9 [12.7; 15.1] (n=70)	0.021
NAC stage			
I; %	59.7 (n=176)	32.4 (n=71)	
II; %	29.0 (n=176)	49.3 (n=71)	<0.001
III; %	11.4 (n=176)	18.3 (n=71)	
Imaging			
LVEF (%); Mdn [Q_1;Q_3]	55 [48; 60] (n=185)	50 [42; 57] (n=75)	0.001
IVSd (mm); M ± SD	18.2±3.2 (n=185)	19.6±3.7 (n=75)	0.005
E/e'; Mdn [Q_1;Q_3]	16 [12; 21] (n=147)	18 [13; 24] (n=66)	0.065
GLS (%); M ± SD	-11.1±3.4 (n=117)	-10.1±3.2 (n=46)	0.083
sPAP (mmHg); Mdn [Q_1;Q_3]	35 [30; 45] (n=130)	38 [30; 45] (n=58)	0.502
TAPSE (mm); Mdn [Q_1;Q_3]	17 [14; 21] (n=175)	15 [12; 20] (n=74)	0.009
Pericardial effusion; %	17.4 (n=184)	29.3 (n=75)	0.048

ACEi = angiotensin-converting enzyme inhibitor; AF = atrial fibrillation; ARB = angiotensin II receptor blocker; ARNI = angiotensin receptor neprilysin inhibitor; ATTR = transthyretin amyloidosis; ATTR-CM = transthyretin amyloid cardiomyopathy; BMI = body mass index; CAD = coronary artery disease; CTS = carpal tunnel syndrome; DBP = diastolic blood pressure; GFR = glomerular filtration rate; GLS = global longitudinal strain; Hb = hemoglobin; HRa = heart rate; hsTnT = high-sensitivity troponin T; IVSd = interventricular septum thickness at end-diastole; LVEF = left ventricular ejection fraction; M = mean; Mdn = median; NAC = National Amyloidosis Centre; NT-proBNP = N-terminal pro-B-type natriuretic peptide; NYHA = New York Heart Association; PM = pacemaker; PNP = polyneuropathy; Q = quartile; SAVR = surgical aortic valve replacement; SBP = systolic blood pressure; SD = standard deviation; sPAP = systolic pulmonary arterial pressure; TAPSE = tricuspid annular plane systolic excursion; TAVR = transcatheter aortic valve replacement; the 'n' within the columns indicates the number of patients for whom the respective variable was available.

Table S2: Univariable and multivariable Cox regression models with hazard ratios for the primary endpoint of all-cause mortality.

	univariable			multivariable		
**Variable**	**HR**	**95% CI**	**p**	**HR**	**95% CI**	**p**
**Age**	**1.076**	**1.030-1.123**	**<0.001**	1.034	0.986-1.084	0.163
**NYHA class**	**2.068**	**1.353-3.161**	**0.001**	**1.787**	**1.115-2.865**	**0.016**
PNP	0.864	0.501-1.490	0.598	1.192	0.635-2.236	0.584
**Serum albumin**	**0.866**	**0.808-0.929**	**<0.001**	**0.871**	**0.797-0.951**	**0.002**
**NAC**	**1.759**	**1.268-2.441**	**<0.001**	1.362	0.926-2.001	0.116
**Tafamidis 61 mg**	**0.233**	**0.142-0.383**	**<0.001**	**0.330**	**0.174-0.626**	**<0.001**

CI = confidence interval; NAC = National Amyloidosis Centre; HR = hazard ratio; NYHA = New York Heart Association; PNP = polyneuropathy.

Table S3: Univariable and multivariable Cox regression models with hazard ratios for the secondary composite endpoint of all-cause mortality and/or hospitalisation due to HF.

	univariable			multivariable		
**Variable**	**HR**	**95% CI**	**p**	**HR**	**95% CI**	**p**
**Age**	**1.074**	**1.042-1.107**	**<0.001**	**1.056**	**1.015-1.098**	**0.007**
**NYHA class**	**1.729**	**1.318-2.267**	**<0.001**	1.394	0.966-2.012	0.076
PNP	0.939	0.642-1.374	0.747	1.143	0.706-1.851	0.585
**Serum albumin**	**0.885**	**0.835-0.938**	**<0.001**	**0.899**	**0.841-0.963**	**0.002**
**NAC**	**1.651**	**1.310-2.081**	**<0.001**	1.291	0.954-1.747	0.098
**Tafamidis 61 mg**	**0.473**	**0.320-0.701**	**<0.001**	0.692	0.403-1.187	0.181

CI = confidence interval; NAC = National Amyloidosis Centre; HR = hazard ratio; NYHA = New York Heart Association; PNP = polyneuropathy.

Table S4: Baseline characteristics of a representative propensity-score matched ATTR-CM cohort (based on imputation 16) stratified by treatment status.

	**ATTR-CM study cohort** n = 141	**Tafamidis 61 mg** n = 94 (66.7%)	**No specific therapy** n = 47 (33.3%)	**p**
Type of ATTR-CM				
wild-type; %	81.6	85.1	74.5	
variant; %	11.3	9.6	14.9	0.277
not tested; %	7.1	5.3	10.6	
Time since initial diagnosis (months); Mdn [Q_1;Q_3]	4 [1; 17]	6 [1; 17]	2 [0; 15]	0.112
≤ 12 months since initial diagnosis; %	67.4	66.0	70.2	0.751
Follow-up time (months); Mdn [Q_1;Q_3]	39 [17; 48]	43 [21; 48]	33 [11; 46]	0.016
Demographic data				
Male patients; %	82.3	85.1	76.6	0.311
Age (years); Mdn [Q_1;Q_3]chi	79 [75; 82]	78 [73; 82]	80 [76; 84]	0.076
Age ≥ 80 years; %	46.1	40.4	57.4	0.083
BMI (kg/m^2); Mdn [Q_1;Q_3]	26.0 [24.2; 29.1]	25.9 [24.1; 29.0]	26.4 [24.5; 29.2]	0.519
Modified BMI [(kg/m^2)x(g/L)]; Mdn [Q_1;Q_3]	1,099 [1,011; 1,207]	1,094 [991; 1,205]	1,110 [1,024; 1,211]	0.603
Vital parameters				
SBP (mmHg); M ± SD	134±20	135±19	133±21	0.574
DBP (mmHg); M ± SD	77±12	77±12	78±13	0.962
HRa (1/min); M ± SD	74±14	73±14	75±14	0.446
Symptoms				
NYHA class				
I; %	17.7	19.1	14.9	
II; %	36.9	37.2	36.2	
III; %	45.4	43.6	48.9	0.793
IV; %	0.0	0.0	0.0	
History of cardiac decompensation; %	30.5	31.9	27.7	0.746
Comorbidities				
History of arterial hypertension; %	65.2	60.6	74.5	0.150
Diabetes mellitus; %	17.0	16.0	19.1	0.812
CAD; %	40.4	39.4	42.6	0.856
AF; %	57.4	58.5	55.3	0.857
History of CTS; %	39.0	42.6	31.9	0.299
Lumbar spinal stenosis; %	10.6	13.8	4.3	0.148
History of SAVR or TAVR %	5.7	4.3	8.5	0.441
PNP; %	31.9	40.4	14.9	0.004
PM; %	15.6	17.0	12.8	0.682
Medication				
Diuretics; %	64.5	64.9	63.8	1.000
ACEi, ARB or ARNI; %	56.0	56.4	55.3	1.000
Beta-blocker; %	54.6	53.2	57.4	0.765
Oral anticoagulation; %	60.3	58.5	63.8	0.670
Biomarker				
Serum albumin (g/L); Mdn [Q_1;Q_3]	42 [40; 44]	43 [41; 44]	42 [40; 44]	0.619
NT-proBNP (pg/mL); Mdn [Q_1;Q_3]	2,977 [1,260; 4,951]	2,989 [1,306; 4,838]	2,837 [719; 5,577]	0.718
GFR (mL/min); Mdn [Q_1;Q_3]	58 [45; 75]	59 [47; 75]	58 [42; 75]	0.791
HsTnT (ng/mL); Mdn [Q_1;Q_3]	0.041 [0.029; 0.063]	0.039 [0.030; 0.068]	0.042 [0.029; 0.060]	0.906
Hb (g/dL); Mdn [Q_1;Q_3]	13.6 [12.7; 14.6]	13.6 [12.5; 14.7]	13.6 [12.9; 14.4]	0.676
NAC stage				
I; %	46.1	45.7	46.8	
II; %	33.3	35.1	29.8	0.760
III; %	20.6	19.1	23.4	
Imaging				
LVEF (%); Mdn [Q_1;Q_3]	54 [45; 60]	54 [45; 60]	55 [45; 60]	0.988
IVSd (mm); M ± SD	18.4±4.0	18.7±3.8	17.8±4.4	0.235
E/e'; Mdn [Q_1;Q_3]	16 [12; 22]	16 [12; 21]	18 [11; 23])	0.543
GLS (%); M ± SD	-10.7±3.5	-10.8±3.1	-10.5±4.1	0.720
sPAP (mmHg); Mdn [Q_1;Q_3]	35 [29; 44]	35 [28; 43]	35 [30; 45]	0.880
TAPSE (mm); Mdn [Q_1;Q_3]	17 [12; 21]	17 [12; 21]	16 [11; 22]	0.801
Pericardial effusion; %	24.1	22.3	27.7	0.626

ACEi = angiotensin-converting enzyme inhibitor; AF = atrial fibrillation; ARB = angiotensin II receptor blocker; ARNI = angiotensin receptor neprilysin inhibitor; ATTR = transthyretin amyloidosis; ATTR-CM = transthyretin amyloid cardiomyopathy; BMI = body mass index; CAD = coronary artery disease; CTS = carpal tunnel syndrome; DBP = diastolic blood pressure; GFR = glomerular filtration rate; GLS = global longitudinal strain; Hb = hemoglobin; HRa = heart rate; hsTnT = high-sensitivity troponin T; IVSd = interventricular septum thickness at end-diastole; LVEF = left ventricular ejection fraction; M = mean; Mdn = median; NAC = National Amyloidosis Centre; NT-proBNP = N-terminal pro-B-type natriuretic peptide; NYHA = New York Heart Association; PM = pacemaker; PNP = polyneuropathy; Q = quartile; SAVR = surgical aortic valve replacement; SBP = systolic blood pressure; SD = standard deviation; sPAP = systolic pulmonary arterial pressure; TAPSE = tricuspid annular plane systolic excursion; TAVR = transcatheter aortic valve replacement; the 'n' within the columns indicates the number of patients for whom the respective variable was available.
